# Standardization of whole blood immune phenotype monitoring for clinical trials: panels and methods from the ONE study

**DOI:** 10.1186/2047-1440-2-17

**Published:** 2013-10-25

**Authors:** Mathias Streitz, Tewfik Miloud, Michael Kapinsky, Michael R Reed, Robert Magari, Edward K Geissler, James A Hutchinson, Katrin Vogt, Stephan Schlickeiser, Anders Handrup Kverneland, Christian Meisel, Hans-Dieter Volk, Birgit Sawitzki

**Affiliations:** 1 Institute of Medical Immunology, Charité - Universitätsmedizin Berlin, Augustenburger Platz 1, Berlin 13353, Germany; 2 Global Assay and Applications Development, Beckman Coulter, Inc, 130 Av. De Lattre de Tassigny, B.P. 177, Marseille Cedex 9, France; 3 Global Assay and Applications Development, Beckman Coulter, Inc, 11800 SW 147th Ave, Miami, FL 33196, USA; 4 Department of Surgery, University Hospital Regensburg, University of Regensburg, Regensburg, Germany; 5 Berlin-Brandenburg Center for Regenerative Therapies (BCRT), Charité - Universitätsmedizin Berlin, Berlin, Germany

**Keywords:** Immune monitoring, Organ transplantation, Cell therapy, Tolerance, Kidney, Flow cytometry

## Abstract

**Background:**

Immune monitoring by flow cytometry is a fast and highly informative way of studying the effects of novel therapeutics aimed at reducing transplant rejection or treating autoimmune diseases. The ONE Study consortium has recently initiated a series of clinical trials aimed at using different cell therapies to promote tolerance to renal allografts. To compare the effectiveness of different cell therapies, the consortium developed a robust immune monitoring strategy, including procedures for whole blood (WB) leukocyte subset profiling by flow cytometry.

**Methods:**

Six leukocyte profiling panels computing 7- to 9-surface marker antigens for monitoring the major leukocyte subsets as well as characteristics of T cell, B cell, and dendritic cell (DC) subsets were designed. The precision and variability of these panels were estimated. The assay was standardized within eight international laboratories using Flow-Set Pro beads for mean fluorescence intensity target definition and the flow cytometer setup procedure. Standardization was demonstrated by performing inter-site comparisons.

**Results:**

Optimized methods for sample collection, storage, preparation, and analysis were established, including protocols for gating target subsets. WB specimen age testing demonstrated that staining must be performed within 4 hours of sample collection to keep variability low, meaning less than or equal to 10% for the majority of defined leukocyte subsets. Inter-site comparisons between all participating centers testing shipped normal WB revealed good precision, with a variability of 0.05% to 30% between sites. Intra-assay analyses revealed a variability of 0.05% to 20% for the majority of subpopulations. This was dependent on the frequency of the particular subset, with smaller subsets showing higher variability. The intra-assay variability performance defined limits of quantitation (LoQ) for subsets, which will be the basis for assessing statistically significant differences achieved by the different cell therapies.

**Conclusions:**

Local performance and central analysis of the ONE Study flow cytometry panel yields acceptable variability in a standardized assay at multiple international sites. These panels and procedures with WB allow unmanipulated analysis of changes in absolute cell numbers of leukocyte subsets in single- or multicenter clinical trials. Accordingly, we propose the ONE Study panel may be adopted as a standardized method for monitoring patients in clinical trials enrolling transplant patients, particularly trials of novel tolerance promoting therapies, to facilitate fair and meaningful comparisons between trials.

## Introduction

Immune monitoring by flow cytometry is crucial in studying effects of novel therapeutics aimed at modulating the immune response. The ONE Study consortium (http://www.onestudy.org) initiated a series of clinical trials to evaluate cell-based immunotherapies as adjunctive immune modulatory agents in kidney transplantation. The aim of the ONE Study is to conduct multicenter assessments of the biological effect of regulatory immune cells on recipient immune responses after transplantation in comparison to an independent clinical reference group trial using standard medication at transplant centers across Europe and the USA. The basic objective of the ONE Study is to condition the allo-specific immune response to promote protolerogenic responses to renal allografts. Different cell types with immune suppressive characteristics have been described, that are capable of modulating the allo-reactive immune response following transplantation [1-4]. Within the ONE Study trials it is planned to test naturally occurring regulatory T cells (nTregs) [4-8], type 1 regulatory T (Tr1) cells [1,9-11], tolerogenic macrophages (Mregs) [2,12,13], and tolerogenic dendritic cells (DCs) [3,14-16] predominantly for their safety in renal transplantation, but also for signs of their ability to prevent biopsy-proven acute rejection and other transplant-related pathologies, as well as their biological effects on the recipient. To be able to compare the effectiveness of these alternative cellular therapeutics, standardization of the immune monitoring assays is critical. Therefore, the ONE Study consortium developed a robust immune monitoring procedure to profile peripheral blood cellular phenotype and function of whole blood (WB) leukocytes based on flow cytometry.

Since the complexity of the immune system requires the measurement of multiple parameters in parallel and the characterization of many cell subsets, flow cytometry has become a very powerful tool for immune diagnostics [17,18]. However, the assay complexity in combination with a diversity of equipment, reagents, and many other pre-analytical factors such as specimen age, staining procedures, compensation, and analytical factors such as subset definition, also increases the variability, particularly when comparing results obtained within different laboratories. The variables that need to be controlled to ensure standardization have been reviewed elsewhere and different models for standardization of sample handling, instrument setup, data acquisition, and data analysis have been proposed [17-20].

Based on these principles we established robust 7- to 9-color panels for leukocyte profiling capturing the characteristics of the normal immune phenotype of different T cell, B cell, and DC subsets, and their activation status. The ONE Study consortium is using WB as a sample matrix to capture differences in relative and absolute cell counts of populations such as neutrophils, plasmablasts, and DCs, which are removed during peripheral blood mononuclear cell (PBMC) preparation and freezing. This approach requires rigorous control of sample collection and timing of sample preparation.

Here we describe the standardization of leukocyte profiling by flow cytometry between eight transplantation centers located in Europe and the USA for application within the ONE Study. By thorough training that requires strict adherence to standard operating procedures (SOPs), centrally defining target channels for all fluorochromes and transferring those to the cytometers at each site, we achieved comparable results with a low inter-site variability of 0.05% to 30%, depending on the frequency of the cell type. This strategy is applicable for use in multicenter clinical trials, and allows detection of relative and absolute changes of nearly all blood leukocyte subsets.

## Methods

All procedures were described in SOPs, and the technical staff at all sites were trained for on-site performance of the SOPs.

### Blood specimen collection

Healthy individuals were recruited from staff and students of Charité - Universitätsmedizin Berlin, Berlin, Germany. Additionally, blood samples from transplant patients enrolled into the reference trial of the ONE Study were collected 3 to 6 months after kidney transplantation.

Written informed consent was obtained from all participants. The study was approved by the Ethics Committee of the Charité - Universitätsmedizin Berlin.

Blood was collected into vacutainers (BD, Heidelberg, Germany) containing EDTA for anticoagulation. Anticoagulated peripheral blood for age-of-blood test and inter-site comparison was stored at 4°C and shipped in 4°C temperature-controlled boxes. For comparative analysis of different preservatives, blood was also collected into Cyto-Chex BCT tubes (Streck labs, Omaha, NE, USA) and stored at 4°C for the indicated times. All consecutive blood samples were collected considering the impact of the circadian rhythm on leukocyte composition and function, as previously reported [21-24], at similar time points during the day (± 1 hour).

### Antibody panel

Fluorochrome-conjugated anti-human monoclonal antibodies were obtained from Beckman Coulter (Marseille, France), except anti-BDCA-2 and anti-BDCA-3, which were obtained from Miltenyi Biotec (Bergisch Gladbach, Germany), and anti-CCR7, which was obtained from R&D Systems (Wiesbaden, Germany). Six panel matrices were defined for 7- to 9-fluorochrome channels. Antibody clones were chosen based on recommendation at the human leukocyte differentiation antigen (HLDA) workshops or published results. The fluorochromes for each antibody were chosen in order to achieve high sensitivity for the detection of dim antigens. Each antibody was titrated based on achieving the highest signal (mean fluorescence intensity (MFI)) for the positive population and the lowest signal for the negative population representing the optimal signal to noise ratio [25]. After optimization, all panels were formulated at Beckman Coulter. Whenever a change of the antibody batch occurred, the new formulation was tested against the old one, and was only accepted when a variability of less than 4% was achieved. Panel matrices are listed in Additional file 1: Figure S1.

### Leukocyte staining

For staining protocol 1, 100 μL of anticoagulated peripheral blood was stained with surface antibodies for 15 minutes at room temperature in the dark prior to lysis and fixation with VersaLyse + 2.5% IOTest fixative solution (Beckman Coulter) for 15 minutes in the dark. Lysed cells were washed twice (PBS, and PBS containing 2% FCS and 0.1% sodium azide) prior to acquisition. Prepared samples for age-of-stain tests were stored at 4°C. For the investigation of the DC subpopulations, staining was done twice in parallel to gain sufficient cell numbers and both samples were combined before acquisition.

For staining protocol 2, for the investigation of B cell subpopulations, 300 μL of anticoagulated peripheral blood was lysed with ammonium chloride (Beckman Coulter) for 12 minutes at room temperature in rotating tubes and washed twice with cold PBS. Lysed samples were stained for 20 minutes at 4°C in the dark, fixed with 2.5% IOTest fixative solution in PBS for 15 minutes in the dark, and washed once with PBS containing 2% FCS and 0.1% sodium azide.

The SOPs describing staining protocol 1 and 2 can be found within Additional file 2: Method S6 and Additional file 3: Method S7.

Cell staining was performed within 30 minutes, or 4 hours and 24 hours after blood collection for the age-of-blood test. Cell staining for inter-site comparisons took place simultaneously at all centers 24 or 30 hours after blood collection.

### Data acquisition

**Figure 1:**
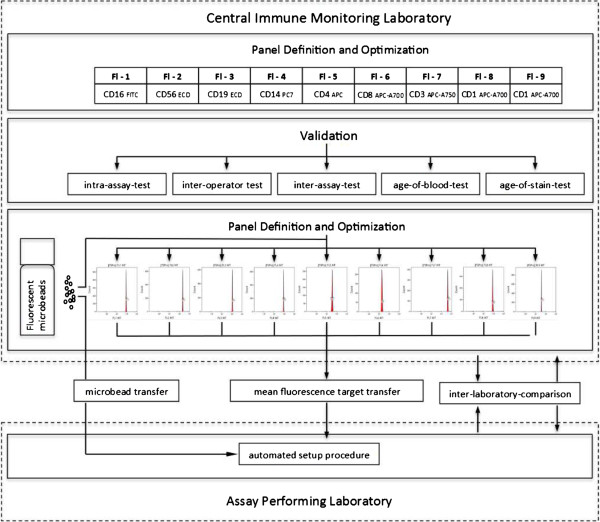
Overview of panel design, standardization, and validation within the ONE-Study.

All samples were measured on 10 color, 3 laser Navios flow cytometers (Beckman Coulter) using two different settings. The first setting was created with anticoagulated peripheral blood samples stained with single antibodies according to staining protocol 1. The second setting was created using pre-lysed blood stained with single antibodies according to protocol 2. Both settings and Navios protocol files were established using a single Navios at the cytometry laboratory of the Medical Immunology at Charité - Universitätsmedizin Berlin. Target channels were defined for all fluorochromes of both settings using calibration bead particles (Flow-Set Pro beads, Beckman Coulter) at the same facility. Protocol files and target channel files were forwarded to all participating flow cytometry facilities (Figure 1). Settings for all Navios cytometers were created using the target files in combination with the auto-setup function of the Navios software and the same lot of calibration bead particles as used for the creation of the settings. The relevant SOPs can be found within the Additional file 2: Method S6 and Additional file 3: Method S7. The same type of Navios flow cytometer was used at all participating sites.

### Validation test design

For the inter-assay test, blood from four healthy individuals was collected and assayed on three consecutive days by two operators. Similarly, for the inter-operator test, blood from three healthy individuals was collected and assayed on two consecutive days by two operators. Within the intra-assay test, the variability in staining results of blood collected from two healthy individuals and assayed five times in parallel by one operator was determined. For the age-of-blood test, blood was taken from six healthy individuals and assayed at three different time points (0 hours, 4 hours, 24 hours) after blood collection by one operator. Similarly, in the age-of-stain test blood was drawn from five healthy individuals, stained in parallel, and measured at three different time points (0 hours, 4 hours, 24 hours) by one operator. To compare the reproducibility of the staining between different sites (inter-site comparison), blood from four healthy individuals was assayed simultaneously at five centers 30 hours after blood collection by different operators. Furthermore, to test whether immunosuppressive therapy may impact variability of leukocyte subset identification, we performed inter-operator tests on samples collected from transplant patients on three different days, which were stained by three different operators.

### Data analysis

All acquired data files were analyzed by the same analyst using the Kaluza software, version 1.2 (Beckman Coulter). Cell doublets were excluded using forward scatter time of flight (wide) versus forward scatter integral (area). Leukocytes were gated using CD45 expression versus side scatter. Absolute counts of the subpopulations were calculated in all panels by use of the CD45^+^ leukocyte ‘backbone’ in combination with the WB count obtained from all samples.

**Figure 2:**
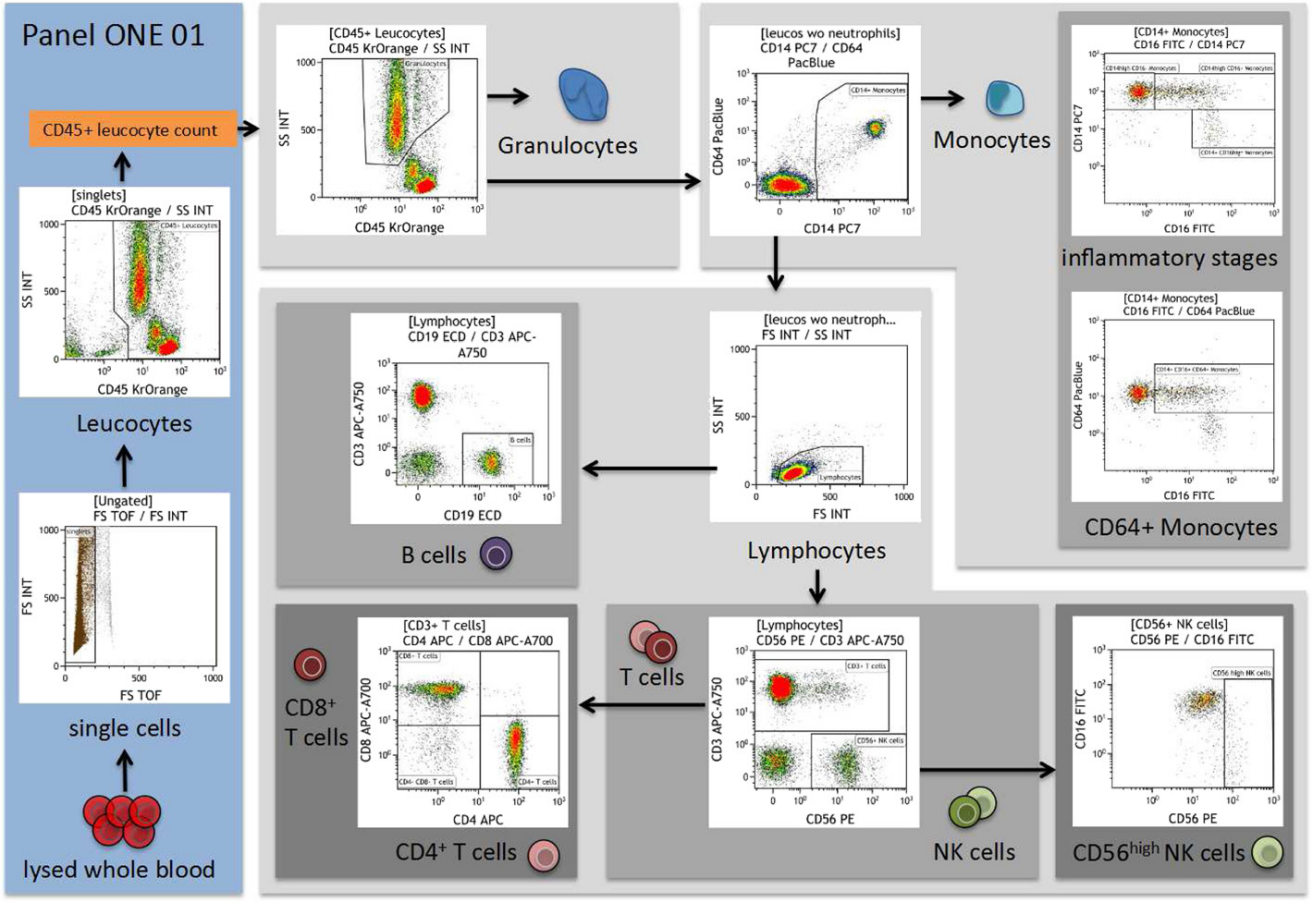
**Overview of the gating strategy for panel ONE 01: general immune phenotype, using the sample of a healthy individual.** The data file of the stained lysed (EDTA spiked) whole blood (WB) was analyzed as follows: exclusion of non-single events (forward scatter time of flight versus forward scatter integral); gating of CD45^+^ leukocytes (anti-CD45 versus sideward scatter integral) – the counted CD45^+^ events were used as the reference for calculating the absolute cell number of indicated populations in WB; gating and exclusion of granulocytes (anti-CD45 versus sideward scatter integral); gating and exclusion of all CD14^+^ monocytes (anti-CD14 versus anti-CD64) – the gated CD14^+^ monocytes were used to further discriminate different inflammatory/differentiation stages of monocytes (anti-CD16 versus anti-CD14) resulting in CD14^++^CD16^-^ classical monocytes, CD14^++^CD16^+^ and CD14^+^CD16^++^ monocytes, and anti-CD16 versus anti-CD64 to capture CD16^+^CD64^+^ monocytes; gating of lymphocytes (forward scatter integral versus sideward scatter integral); gating of CD56^+^NK cells, which were further subdivided into CD56^dim^ and CD56^high^NK cells; gating of CD3^+^ T cells (anti-CD56 versus anti-CD3) – gated T cells were used for identification of CD4^+^ T-cells and CD8^+^ T-cells (anti-CD4 versus anti-CD8), and the gated lymphocytes were also used for identification of the B cell population (anti-CD19 versus anti-CD3). WB, whole blood.

**Figure 3:**
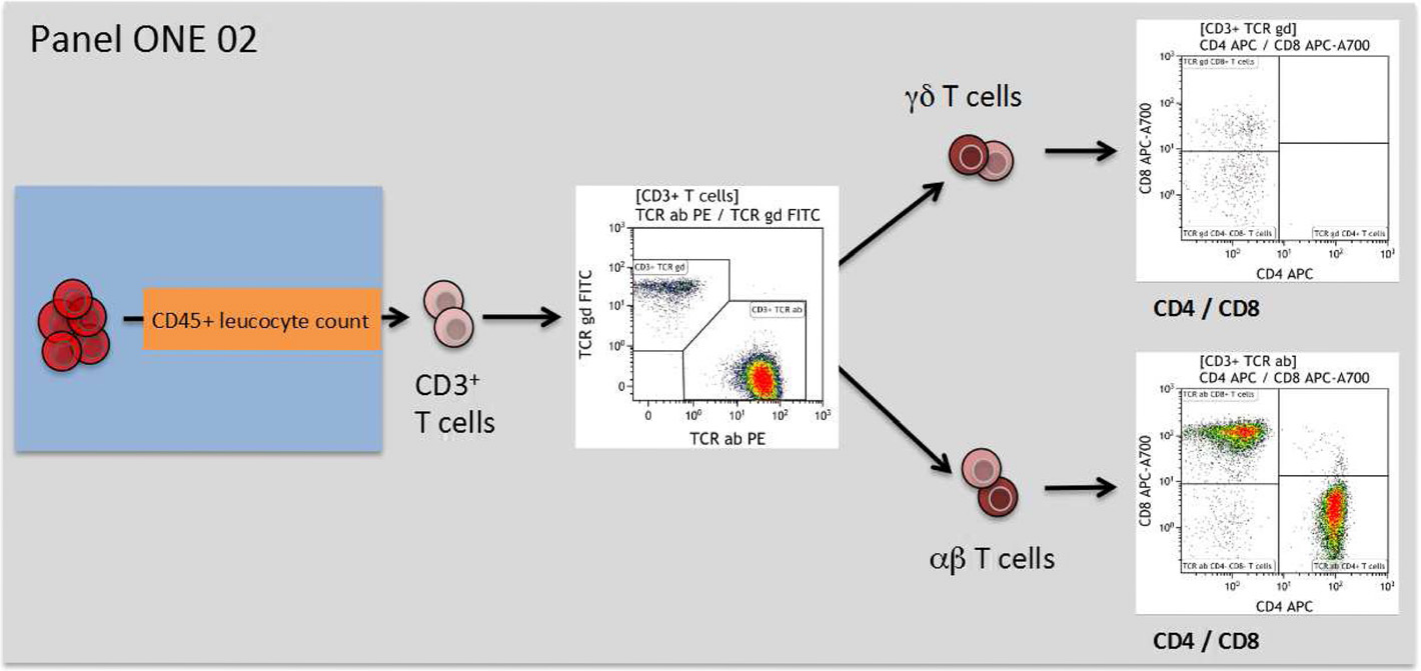
**Overview of the gating strategy for panel ONE 02: T cell subsets/αβ**^**+ **^**T cells and γδ**^**+ **^**T cells.** The data file of the stained lysed (EDTA spiked) whole blood (WB) was analyzed as follows: exclusion of non-single events and gating of CD45^+^ leukocytes as shown for panel ONE 01 (Figure 2); gating of CD3^+^ T cells (anti-CD3 versus sideward scatter); gating of αβ^+^ T cells and γδ^+^ T cells (anti-T cell receptor αβ^+^ T cells versus anti-T cell receptor γδ^**+**^); and gating of CD4^+^ and CD8^+^ T cells for both T cell receptor subsets (anti-CD4 versus anti-CD8). WB, whole blood.

**Figure 4:**
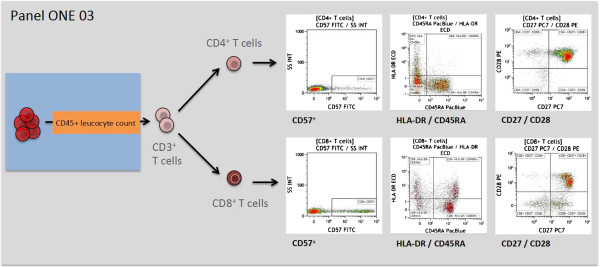
**Overview of the gating strategy for panel ONE 03: T cell activation.** Expression of CD57 or HLA-DR and loss of CD27 or CD28 expression was used as a sign of T cell activation, as previously described [27-32]. The data file of the stained lysed (EDTA spiked) whole blood (WB) was analyzed as follows: exclusion of non-single events and gating of CD45^+^ leukocytes as shown for panel ONE 01 (Figure 2); gating of CD3^+^ T cells (anti-CD3 versus sideward scatter); and gating of CD4^+^ as well the CD8^+^ T cells (anti-CD4 versus anti-CD8), for both subsets gating on CD57^+^ cells (anti-CD57 versus sideward scatter), HLA-DR^+^/CD45RA^+^ (naive, and HLA-DR^+^/CD45RA^-^ (memory), and CD27^-^/^+^ and CD28^-^/^+^ subsets (anti-CD27 versus anti-CD28). WB, whole blood.

**Figure 5:**
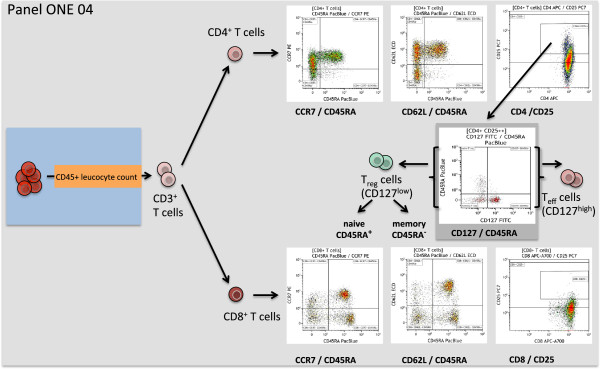
**Overview of the gating strategy for panel ONE 04: memory T cells and regulatory T cells.** The data file of the stained lysed (EDTA spiked) whole blood (WB) was analyzed as follows: exclusion of non-single events and gating of CD45^+^ leukocytes as shown for panel ONE 01 (Figure 2); gating of CD3^+^ T cells (anti-CD3 versus sideward scatter); gating of CD4^+^ as well the CD8^+^ T cells (anti-CD4 versus anti-CD8), for both subsets gating of naive (CCR7^+^ or CD62L^+^ and CD45RA^+^), central memory (CCR7^+^ or CD62L^+^ and CD45RA^-^), effector memory (CCR7^-^ or CD62L^-^ and CD45RA^-^), and TEMRA (CCR7^-^ or CD62L^-^ and CD45RA^+^) subsets, as reported recently [33,34]. CD4^+^CD25^++^ were further separated into CD127^low^ regulatory T cells, discriminating CD45RA^+^ naive and CD45RA^-^ memory regulatory T cells, and CD127^high^ activated effector T cells [35]. We also enumerated activated CD8^+^CD25^++^ cells. WB, whole blood.

**Figure 6:**
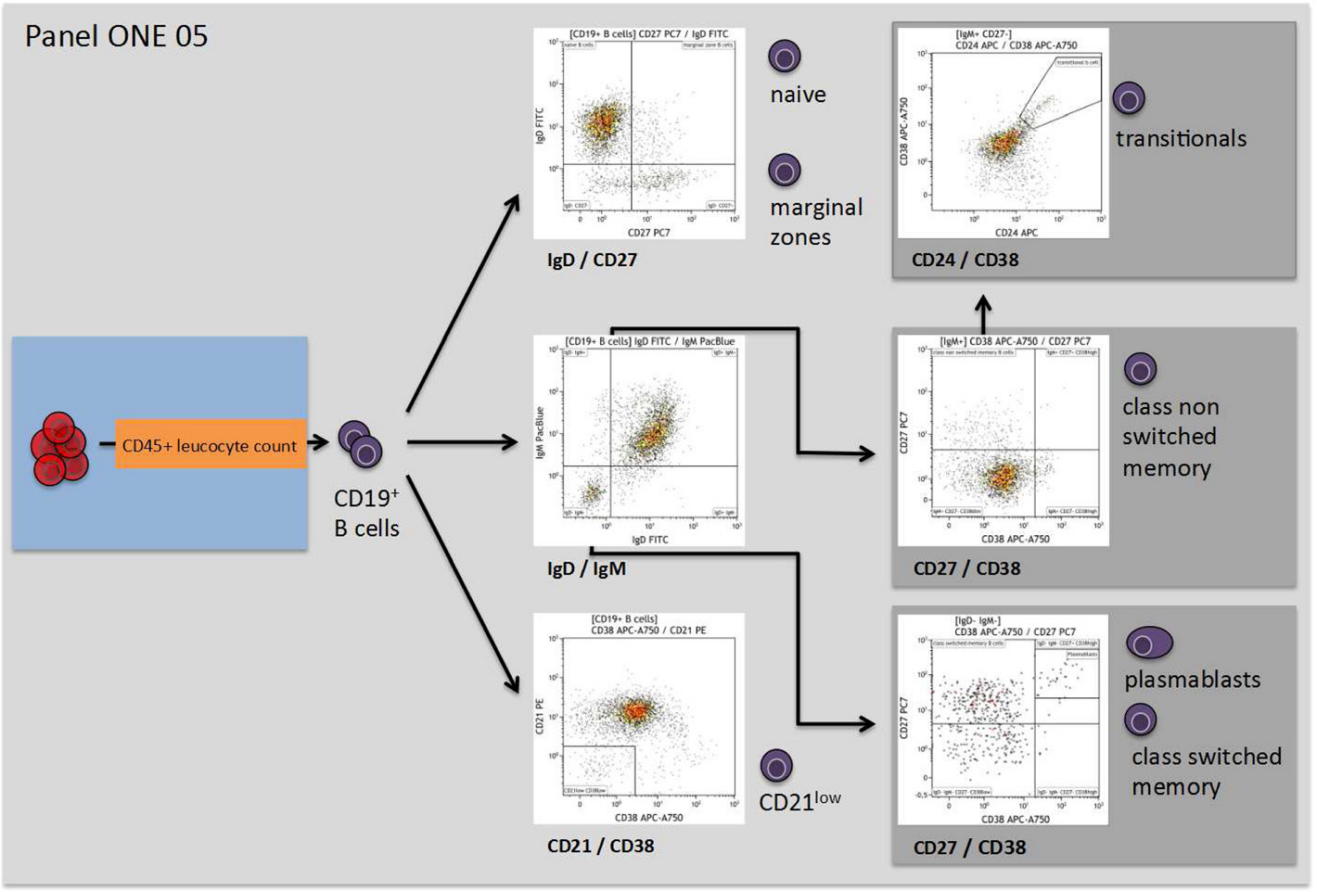
**Overview of the gating strategy for panel ONE 05: B cell subsets.** Identification of B cell subsets was based on previously published classifications [36,37]. The data file of the stained lysed (EDTA spiked) whole blood (WB) was analyzed as follows: exclusion of non-single events and gating of CD45^+^ leukocytes as shown for panel ONE 01 (Figure 2); gating of CD19^+^ B cells (anti-CD19 versus sideward scatter); gating of CD21^low^ B cells (anti-CD38 versus anti-CD21); gating of IgD^-^IgM^-^ and IgM^+^ B cells (anti-IgD versus anti-IgM). Pre-gated IgD^-^IgM^-^ B cells were further used to identify plasmablasts (CD27^+^CD38^high^) and class-switched memory B cells (CD27^+^CD38^low^), pre-gated IgM^+^ B cells were used to identify of class non-switched memory B cells (CD27^+^CD38^low^), and the pre-gated IgM^+^CD27^-^ B cells were used to identify transitional B cells (CD24^+^CD28^high^). WB, whole blood.

**Figure 7:**
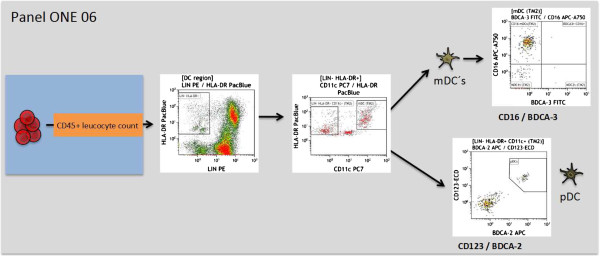
**Overview of the gating strategy for panel ONE 06: dendritic cell (DC) subsets.** DCs and their subpopulations were identified, as previously reported [38-41]. The data file of the stained lysed (EDTA spiked) whole blood (WB) was analyzed as follows: exclusion of non-single events and gating of CD45^+^ leukocytes as shown for panel ONE 01 (Figure 2); gating of lineage (LIN; anti-CD3, anti-CD14, anti-CD19, anti-CD20, anti-CD56) negative HLA-DR^+^ cells, identification of LIN^-^HLA-DR^+^CD11c^+^ myeloid DCs (mDCs), and LIN^-^HLA-DR^+^CD11c^-^ cells (anti-CD11c versus anti-HLA-DR). Pre-gated mDCs were used to identify CD16^+^, mDC1, and BDCA3^+^mDC subsets, and pre-gated LIN^-^HLA-DR^+^CD11c^-^ cells were used to identify plasmacytoid DCs (CD123^+^BDCA2^+^). DC, dendritic cell; mDC, myeloid dendritic cell; LIN, lineage; WB, whole blood.

The cell subset definition and the choice of markers and dyes were discussed and defined within the ONE Study consortium and with Beckman Coulter. The consensus regarding the applied markers defined the gating strategy for the data analysis. To ensure correct identification of negative and positive cell populations, cells were plotted using color density bi-exponential displays, as suggested by Herzenberg *et al.*[26], except for the forward and sideward scatter. See also Figures 2, 3, 4, 5, 6, and 7 for the detailed gating strategy of the different subpopulations.

For the analysis of the inter-assay test, intra-assay test, and inter-site comparison, a template analysis protocol was created for each sample and panel. Data files of the same patient and panel were analyzed by copying the data files of each time point (inter-assay test) or parallel staining (intra-assay test, inter-site comparison) into the appropriate template. Only the sideward scatter (SSC) parameter was adjusted when necessary for the 24- and 30-hour specimens.

Parameters were exported for the calculation of the size and frequencies of the subpopulations from the Kaluza software to Excel (Microsoft, Redmond, WA, USA). The precision profile approach was used to characterize the repeatability performance throughout the range of measurements [42]. Profile is a mathematical function that describes the relationship between the imprecision of the assay and the measuring range. Means and coefficients of variation (CVs) were calculated for each sample. Means of different samples represented the measuring range, while CV represented the corresponding repeatability performance. The relationship between mean and CV throughout the range of measurements was modeled as the repeatability precision profile. A power function was used to model the profiles. The choice of this function was based on the best fit of the data as well as the behavior of the cell counting at different ranges. The shape of the power function was determined by two parameters ‘a’ and ‘b’ that were estimated from the data. The model for the profiles was:

$$CV_{i}=a\;\mathrm{Mea}n_{i}{}^{b}+e_{i}$$

where ‘CV_i_’ and ‘Mean_i_’ were the coefficient of variation and mean for each i-th sample, ‘a’ and ‘b’ were the parameters of the model, and ‘e_i_’ was the random error. The Gauss–Newton method provided in PROC NLIN of SAS/STAT 9.3 (SAS Institute, Cary, NC, USA) was used to obtain the nonlinear least squares estimates of the parameters and their standard errors.

**Figure 8:**
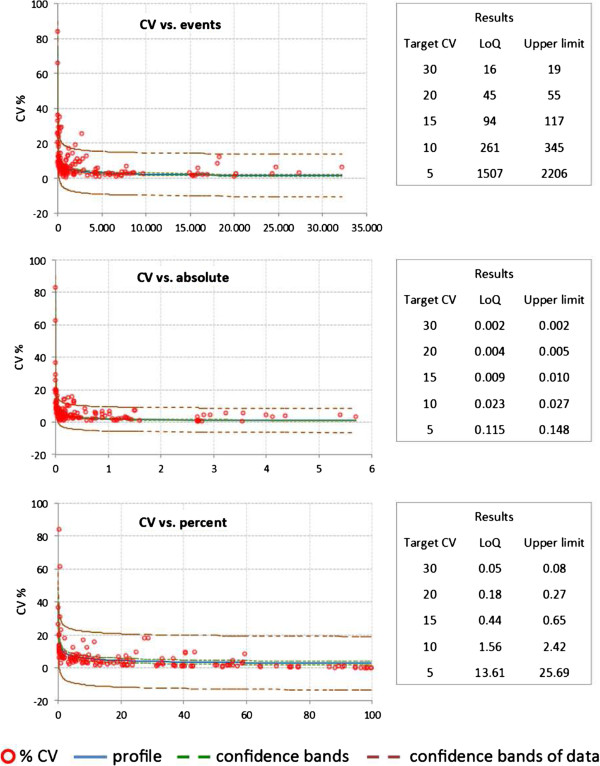
**Single CV values of all cell subsets tested within intra-assays (whole blood (WB) material from two healthy individuals; 71 single subsets = 142 data points).** CV values include five replicates assayed in parallel. Shown are the function, regression, and 95% confidence interval of the CV versus counted events, CV versus calculated absolute cell number of the gated subpopulations, and CV versus percentage of the gated subpopulation. Also shown are the calculated lower limits of quantitation (LoQ) and the upper LoQ for a given CV. CV, coefficient of variation; LoQ, limit of quantitation; WB, whole blood.

Precision profile was used to evaluate the lower limits of the measuring range. We will refer to this limit as the lower limit of quantitation (LoQ) [43]. LoQ was the lowest limit of the measuring range where the leukocyte subpopulation was determined within an acceptable level of imprecision. We chose to represent imprecision by different target CV values, as shown in Figure 8.

The solution for estimating LoQ from the precision profile using different target CV values (Target_CV) was as follows:

$$\mathrm{LoQ}=\exp\left[\frac{\mathrm{Ln}\left(\frac{T\arg\;\mathrm{et}\_\mathrm{CV}}{a}\right)}{b}\right]$$

Taylor’s series approximation was used to calculate standard error of LoQ. The upper confidence limits were based on the standard error of LoQ and 95% confidence interval.

## Results

### Panel setup and optimization of staining procedure

The immune response towards allogeneic transplants involves activation and alterations of leukocytes from both the adaptive and the innate immune system [44-48]. Furthermore, comparative analysis of peripheral blood from operationally tolerant and non-tolerant patients has revealed differences in the composition of, for example, regulatory T cells, memory B cells, but also γδ T cells and NK cells [44,49-52]. Thus, we designed six 7- to 9-color flow panels that allow the capture of frequencies, numbers, differentiation, and activation of nearly all described blood leukocyte subsets (see also Figures 2, 3, 4, 5, 6, and 7, and Additional file 1: Figure S1).

To avoid alterations in leukocyte composition and activation, which can occur upon PBMC isolation, and to ensure comparability between centers, staining was performed on WB and the staining procedure was kept as simple as possible. This resulted in a single protocol for surface staining and for setting compensation of five of the six staining panels, as outlined in the Methods section.

Examination of B cell subpopulations in panel ONE 05 required that free immunoglobulins in the plasma were removed by washing prior to staining of surface IgD and IgM. This washing step was combined with pre-staining erythrocyte lysis, which also ensured the enrichment and subsequent staining of low abundance cells such as plasmablasts and transitional B cells. The pre-washing step was necessary as free immunoglobulins in the serum particularly affect surface staining of IgM and IgD, as shown in Additional file 4: Figure S2.

The gated defining negative and positive leukocyte subpopulations for each surface marker were set using color density bi-exponential displays, as also recommended by others [26], except when displaying the cells according to their scatter signals. All together, the staining procedures described here and the presented gating strategy (Figures 2, 3, 4, 5, 6, and 7) allowed for good discrimination of all target leukocyte populations using fresh WB material of healthy individuals.

### Precision, robustness, and biological variability

**Figure 9:**
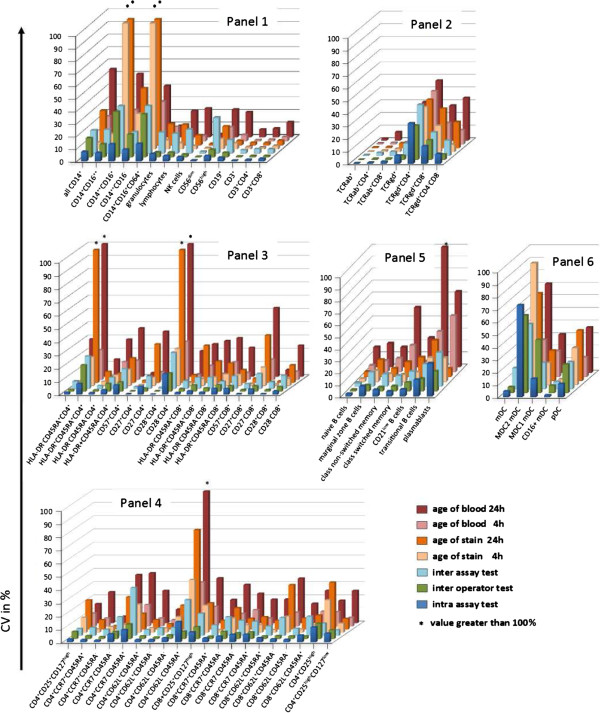
**Mean CVs of cell subsets tested in intra-assay test, inter-operator-test, and inter-assay test, and also the change from baseline for the age-of-stain test 4 hours + 24 hours and the age-of-blood test 4 hours + 24 hours for all six panels.** Panel ONE 01, general immune status; panel ONE 02, T cell subsets/αβ^+^ T cells and γδ^+^ T cells; panel ONE 03, T cell activation; panel ONE 04, T cell memory and regulatory T cells; panel ONE 05, B cell subsets; and panel ONE 06, dendritic cell (DC) subsets. CV, coefficient of variation; DC, dendritic cell.

To evaluate the precision of the test system, intra-assay, inter-assay, and inter-operator tests were performed, and the CVs of percent positive cells and absolute cell counts were calculated. A fixed template for gating on leukocyte subsets and defining true positive subpopulations was used to improve standardization of the analysis system. The CV for the intra-assay test with five assay replicates per WB sample allowed an analysis of the tube-to-tube consistency. The replicate tube was assayed in parallel, and the intra-assay CV was calculated from the relative values, the counted event numbers, and the absolute cell number for each population (Figure 9).

At least 142 single CVs of 71 subpopulations were included in the analysis (Figures 8 and 9). Comparing the relative counts for the gated populations (that is, percentages), only three cell populations from the 71 total populations exceeded a CV of 20%. Furthermore, only 12 cell populations had a CV of greater than 10%. Similar results were obtained when calculating the CV for absolute cell numbers. Five populations showed a CV above 20%, and 20 populations a CV above 10%. CV values calculated for the percentages of all lineage markers (CD45, CD3, CD4, CD8, CD19, CD56) were below 4%, except for the monocytes, which had a mean CV of 7.5%.

**Figure 10:**
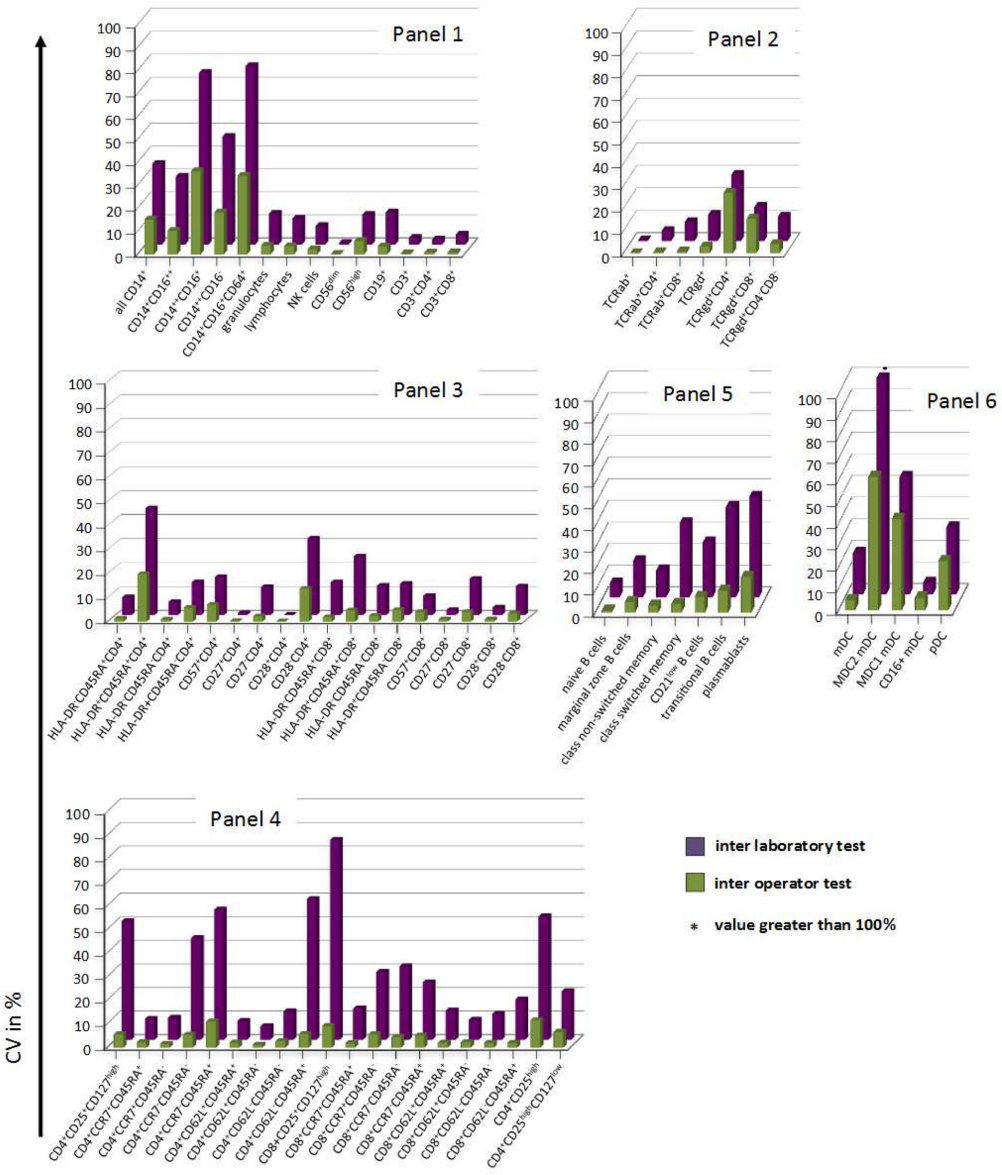
**Mean CVs of cell subsets tested in inter-operator test and inter-laboratory test for all six panels.** Panel ONE 01, general immune status; panel ONE 02, T cell subsets/αβ^+^ T cells and γδ^+^ T cells; panel ONE 03, T cell activation; panel ONE 04, T cell memory and regulatory T cells; Panel ONE 05, B cell subsets; and panel ONE 06, dendritic cell (DC) subsets. CV, coefficient of variation; DC, dendritic cell.

The inter-operator CV was calculated as outlined for the intra-assay test. Mean CVs were compared to the calculations of the intra-assay test; more than 80% of the CVs varied by less than 1% (Figure 10). Considerable increases in inter-operator CVs, as compared to intra-assay CVs, were observed for the monocytes and their subpopulations, which was independent of the size of the populations or the number of counted events. To test whether immunosuppressive therapy may impact variability of leukocyte subset identification, we performed inter-operator tests on samples collected from transplant patients on three different days, which were stained by three different operators. Importantly, all specified leukocyte subpopulations could be identified in the blood of immunosuppressed transplant patients. Furthermore, the determined variability of individual subsets was nearly identical as compared to values detected by staining blood collected from healthy volunteers (Additional file 5: Figure S3).

Inter-assay variability was determined by staining cells from four healthy individuals on three consecutive days. The mean CVs of the inter-assay test were up to 12% greater than the CVs of the intra-assay for the majority of cell populations (Figure 9). This is consistent with previous observations that the biological variability in a WB test with fresh blood donations over several consecutive days is significantly greater than classical inter-assay variability, at which the same sample is stained on three consecutive days [46]. For some leukocyte subsets such as CD4^+^CD28^-^ T cells, plasmablasts, or MDC2 DCs, higher CVs (up to 30%) were observed due to the small population size (lower event counts). Only the CV values for the monocyte subpopulations increased similarly to the inter-operator test, regardless of population size. Stabilizing blood collection devices (for example, Cyto-Chex blood collection tubes) could not be used to reduce inter-assay variability, as they affect expression of some surface markers such as CD62L and CCR7 on T cells, and particularly IgM on B cells (Additional file 6: Figure S4). Therefore, inter-assay data was excluded from the precision and LoQ estimations, as described later on (Figure 8).

### Stability of collected material and stained samples

The variability as a result of blood specimen age prior to staining was estimated by the age-of-blood test. The variability for a change from baseline (0 hours) of 4 hours ranged up to 65% for some gated populations, compared to immediate staining after blood collection. Again, variability was highest in low-abundant cell populations such as plasmablasts (CV of 65%). Thus the change from baseline variability negatively correlates with the WB frequency or absolute cell count.

Within the 4-hour window, a small amount of variability was observed for all lineage markers and T cell subsets (up to 10%), whereas the variability for the monocyte, B cell, and DC subpopulations increased by up to more than 20%. Performing the staining at 24 hours after blood collection increased the variability for all subsets, especially for the aforementioned critical populations, which in some cases reached excessive values of up to 200%. Thus, staining of WB samples within 4 hours of collection is of utmost importance for reliable results.

Next, we determined the variability in defining the leukocyte subsets following a time delay of 4 hours between sample staining and measurement. We observed very little variability with small CVs for most parameters, which again correlated with the number of counted events and percentages of gated populations (Figure 9). Variability of infrequent populations increased slightly following a time delay of 24 hours between staining and measurement. Significant differences were found again only for monocyte subpopulations.

### Reproducibility test by inter-site comparison

To evaluate the reproducibility between participating sites, inter-site comparisons were performed. Different time points for the first enrollment of patients at different ONE Study sites meant that this test was performed several times, but always included different participating sites. Here we show the results of two inter-site comparisons between three laboratories within Europe for the first comparison, and between five laboratories in Europe and one in the USA for the second comparison. The second inter-laboratory comparison posed a particular challenge due to the long shipment time of WB samples from Berlin to San Francisco. The specimen age stability data described above meant that there was an expected impact due to the age of the blood specimen. Assays were performed simultaneously according to the standard SOPs at all sites. Due to shipment, staining was started 24 hours after blood collection for the first inter-site comparison and 30 hours after blood collection in case of the second test. Overall CVs below 15% were obtained for 66% of the parameters (Figure 10). Similar to the other variability tests, significant increases were observed in the CVs for the monocyte subpopulations, and rather infrequent B cell (transitional B cells and plasmablasts), DC, and CD4^+^CD25^high^ T cell populations.

The original data of all validation tests are given within Additional file 7: Figure S5.

### Defining limits of quantitation (LoQ)

Since the biological variability of blood collected on three consecutive days, as it was done for the inter-assay variability test, can be quite high and does not correspond to a classical inter-assay test, the results of the intra-assay evaluation were used to calculate the LoQ and 95% confidence interval (see Methods section).

When plotting CVs versus the percentages, absolute numbers and counted events for each leukocyte subpopulation revealed increasing CVs as a function of all three parameters (Figure 8). We detected increased CV values with a decreased number of counted events, absolute cell numbers, and acquired numbers of cells in leukocyte subpopulations. Therefore, it is impossible to define the same cut-off value for the CV of all leukocyte subsets. We rather calculated the lower (LoQ) and upper limits of absolute cell counts, frequencies, or acquired events for a given target CV using the power function described within the Methods (Figure 8). Accordingly, a leukocyte subpopulation occurring with a frequency of 0.5% per all CD45^+^ leukocytes can be identified and quantified assuming a CV of up to 15%. All subpopulations met these LoQ criteria except for two monocyte subpopulations (CD16^+^CD64^+^ and CD14^high^CD16^+^), and the CD4^+^CD25^high^ subpopulation. This function of CVs will also ensure a reliable definition of significant differences observed for a subset between patient populations. Changes in leukocyte subsets between patient populations will need to be equal or higher to the corresponding CV calculated by the function.

## Discussion

As the precision of flow cytometric profiling of WB samples is impacted by many variables, standardization becomes crucial for use when immune monitoring within clinical trials. For the immune monitoring of transplant patients within the ONE Study, we defined six flow cytometry panels that are being used to stain WB samples locally at all participating clinical sites, followed by a central gating and leukocyte subset analysis, as previously recommended [17,19,26,53]. The results of the performance evaluation of the panels described here demonstrate highly reproducible SOPs that are applicable in general for use in multicenter clinical trials.

We detected low variability in leukocyte subset frequency and absolute counts between different local laboratories for the majority of the leukocyte cell subpopulations, especially for the T cell subsets. Although more variable, monocyte subsets and certain subsets of DCs and B cells show acceptable variability of up to 30%. While the affected DC and B cell subsets encompass small populations with low absolute cell counts that contribute to higher variability, monocyte subsets show higher variability independent of population size or absolute cell count. Nonetheless, we are now able to define limits for statistically significant relative and absolute changes between patient cohorts for the leukocyte subsets as defined by intra-assay (Figures 8 and 9) and inter-operator variability, which is highly dependent on the subset abundance, as previously reported [53]. Applying the developed function for target CVs in relation to, for example, cell frequency, observed differences for a leukocyte subset between two patient populations can only reach significance if those differences are higher than the calculated CV. Thus, a leukocyte population occurring on average at a frequency of 0.5% can only change significantly if an increase or decrease by at least 15% is detected.

Importantly, the acceptable variability in this study was not only achieved when staining samples collected from healthy volunteers, but also with samples from kidney transplant recipients on immunosuppressive therapy. This clearly shows the feasibility of the staining protocols for use in multicenter clinical trials enrolling kidney transplant patients.

There are many possible reasons for exceptional behavior of monocytes and their subsets, and difficulties in standardized enumeration of monocytes have been reported previously [54,55]. Due to their inherent nature, monocytes tend to adhere to plastic surfaces, which may become more relevant with a longer storage time. However, while this can explain the variability observed due to specimen age and inter-site comparisons with aged (24- and 30-hour) blood, it does not explain the increased variability detected within inter-assay and inter-operator comparisons.

Considering all factors, including the feasibility of a minimal time delay between blood collection/processing, and our observed alterations in determined subset frequency and count, we defined a time window of 4 hours for WB staining. By doing so, an acceptable variability of below 30% is achievable for most cell populations, except for infrequent populations such as plasma blasts or certain monocyte subpopulations.

It has been reported by several groups that perhaps the largest single contributor to variability in flow cytometry is the difference in gating the target leukocyte populations [17-19,56]. To control for such alteration, a centralized gating and target definition strategy was chosen. This strategy corresponds to the ‘mixed model’ reported by Maecker *et al*. [17], in which samples are supposed to be obtained, processed, and acquired at local sites through the use of strict SOPs, with a central laboratory performing the analysis of acquired flow cytometry files.

To avoid misclassification of negative and positive leukocyte subsets, and thus misinterpretation of the results, which can easily happen within multicenter clinical trials, we based the gating strategy nearly completely on color density bi-exponential displays. Furthermore, following panel establishment and optimization, a template analysis protocol for the gating strategy was created for each panel, approved by all sites, and subsequently used for the analysis of all files. Only in the case of the 24-hour age-of-blood and inter-site comparisons did the pre-defined gates have to be slightly modified. This was due to alterations in forward and side scatter positions of individual leukocyte subsets upon prolonged WB storage (24 and 30 hours in case of inter-site comparisons). During the trial, specimen age will be strictly controlled to less than 4 hours before staining, so this gating change will not be needed and will not impact patient sample analysis.

In summary, our defined flow cytometry panels allow enumeration of the abundance and activation of a large number of leukocyte subsets with a high precision across multiple international sites. Using this standardized strategy of leukocyte profiling to identify changes in leukocyte subsets, we propose that it will be feasible to detect effects of immunomodulatory treatments within and between multicenter clinical trials. Therefore, this operating procedure will not only be useful in the context of the ONE Study trials, it could also be applied to other clinical trials where sensitive immune monitoring provides valuable information. Equally important, the sharing of procedures and protocols for flow cytometry will allow for more reliable comparisons of immunological effects between these different clinical trials.

## Abbreviations

CV: Coefficient of variation; DC: Dendritic cell; EDTA: Ethylenediaminetetraacetic acid; FCS: Fetal calf serum; HLDA: Human leukocyte differentiation antigen; IgD: Immunoglobulin D; IgM: Immunoglobulin M; LoQ: Limit of quantitation; mDC: Myeloid dendritic cell; MFI: Mean fluorescence intensity; Mreg: Regulatory macrophage; NK: Natural killer; nTreg: Naturally occurring regulatory T cell; PBMC: Peripheral blood mononuclear cell; PBS: Phosphate buffered saline; SOP: Standard operating procedure; SSC: Sideward scatter; Tr1: Type 1 regulatory T; WB: Whole blood.

## Competing interests

Reagents and Navios flow cytometers for this study were provided by Beckman Coulter. MK, RM, TM, and MR are employees of Beckman Coulter.

## Authors’ contributions

MS, TM, MK, MR, EKG, JH, CM, HDV, and BS participated in research design. MS, RM, and BS participated in writing of the manuscript. MS, TM, MK, KV, SS, and AHK participated in the performance of the research. MS, RM, and BS participated in the data analysis. All authors read and approved the final manuscript.

## Supplementary Material

Additional file 1: Figure S1 Panel matrix for flow cytometry-based immune monitoring within the ONE Study. Listed are all antibodies with clone ID and fluorochrome conjugate. Click here for file

Additional file 2: Method S6 Method describing the standard operating procedure for staining of whole blood leukocytes with panel ONE 01, 02, 03, 04, 06. Click here for file

Additional file 3: Method S7 Describing the standard operating procedure for staining of whole blood leukocytes with panel ONE 05. Click here for file

Additional file 4: Figure S2 Exemplary dot plots for surface IgM staining on CD19^+^ B cells comparing whole blood (WB) staining with and without prior removal of free plasma immunoglobulins by an ammonium chloride-based lyse/wash step. Click here for file

Additional file 5: Figure S3 Mean CVs of cell subsets were calculated in inter-operator test on samples collected from transplant patients 3 to 6 months after kidney transplantation for all six panels: panel ONE 01, general immune status; panel ONE 02, T cell subsets/αβ^+^ T cells and γδ^+^ T-cells; panel ONE 03, T cell activation; panel ONE 04, T cell memory and regulatory T cells; panel ONE 05, B cell subsets; and panel ONE 06, dendritic cell (DC) subsets. Click here for file

Additional file 6: Figure S4 Comparative analysis of leukocyte staining of whole blood (WB) samples collected into EDTA or Cyto-Chex tubes. Shown are dot plots of CCR7 versus CD45RA, and CD62L versus CD45RA staining for CD4^+^ T cells using the same gating strategy as described in Figure 5. Additionally IgM versus IgD staining of CD19^+^ B cells is displayed applying the same gating strategy as described in Figure 6. Click here for file

Additional file 7: Figure S5 Shown are all results for the validation of the flow cytometry immune monitoring for the ONE Study, including all single CVs and mean CVs, respectively, and all changes from baseline and mean changes from baseline for all test assays. Click here for file
