# Supplementary material for: Standardization of whole blood immune phenotype monitoring for clinical trials: panels and methods from the ONE study
Source: Transplant Res. 2013 Oct 25;2:17. doi: 10.1186/2047-1440-2-17 (PMC3827923; doi:10.1186/2047-1440-2-17)
Supplement: Additional file 1: Figure S1 — Panel matrix for flow cytometry-based immune monitoring within the ONE Study. Listed are all antibodies with clone ID and fluorochrome conjugate. [file 2047-1440-2-17-S1.pdf]

**Supplementary Figure 1**

| Fluorochrome | leucocyte profile | T cell receptor subsets | T cell activaiton | Teff/mem + Treg | B cell phenotyping | Dendritic cells |
|---|---|---|---|---|---|---|
| FITC | CD16<br>*Clone 3G8* | TCRgd<br>*Clone IMMU510* | CD57<br>*Clone NC1* | CD127<br>*Clone R34.34* | IgD<br>*Clone IADB6* | BDCA3<br>*Clone 501733* |
| PE | CD56<br>*Clone N901* | TCRab<br>*Clone IP26A* | CD28<br>*Clone CD28.2* | CCR7<br>*Clone 150503* | CD21<br>*Clone BL13* | LIN |
| ECD | CD19<br>*Clone J3-119* | CD45RO<br>*Clone UCHL1* | HLA-DR<br>*Clone IMMU357* | CD62L<br>*Clone DREG56* | CD19<br>*Clone J3-119* | CD123<br>*Clone SSDCLY107D2* |
| PC7 | CD14<br>*Clone RMO52* | | CD27<br>*Clone 1A4CD27* | CD25<br>*Clone B1.49.9* | CD27<br>*Clone 1A4CD27* | CD11c<br>*Clone BU15* |
| APC | CD4<br>*Clone 13B8.2* | CD4<br>*Clone 13B8.2* | CD4<br>*Clone 13B8.2* | CD4<br>*Clone 13B8.2* | CD24<br>*Clone ALB9* | BDCA2<br>*Clone AC144* |
| A700 | CD8<br>*Clone B9.11* | CD8<br>*Clone B9.11* | CD8<br>*Clone B9.11* | CD8<br>*Clone B9.11* | | |
| APC-A750 | CD3<br>*Clone UCHT1* | CD3<br>*Clone UCHT1* | CD3<br>*Clone UCHT1* | CD3<br>*Clone UCHT1* | CD38<br>*Clone LS198-4-3* | CD16<br>*Clone 3G8* |
| PacBlue | CD64<br>*Clone 22* | | CD45RA<br>*Clone 2H4LDH11LDB9* | CD45RA<br>*Clone 2H4LDH11LDB9* | IgM<br>*Clone SA-DA-4* | HLA-DR<br>*Clone IMMU357* |
| KrOrange | CD45<br>*Clone J.33* | CD45<br>*Clone J.33* | CD45<br>*Clone J.33* | CD45<br>*Clone J.33* | CD45<br>*Clone J.33* | CD45<br>*Clone J.33* |
