# Supplementary material for: Standardization of whole blood immune phenotype monitoring for clinical trials: panels and methods from the ONE study
Source: Transplant Res. 2013 Oct 25;2:17. doi: 10.1186/2047-1440-2-17 (PMC3827923; doi:10.1186/2047-1440-2-17)
Supplement: Additional file 2: Method S6 — Method describing the standard operating procedure for staining of whole blood leukocytes with panel ONE 01, 02, 03, 04, 06. [file 2047-1440-2-17-S2.pdf]

Instruments

NAVIOS, Beckman Coulter
KALUZA analysis software, Beckman Coulter
Centrifuge
Vortexer
Refrigerator
Pipettes
Pipette boy

Materials

Pipette tips (5 µl - 1000 µl) for low volume (µl) pipettes
Pipette tips for pipette boy
12x75mm-Flow cytometry tubes
Rack for 12x75mm-tubes

Name	Company	Order no.
IsoFlow™	Beckman Coulter	8546859
Versa Lyse™ – Lysing solution	Beckman Coulter	A09777
IOTest® 3 Fixative solution (10x)	Beckman Coulter	A07800
Test tube, 12 x 75 mm Blue, 250 pieces	Beckman Coulter	2523749
FCS – head inactivated<br>or an other comparable commercial product	Biochrom	S0115
NaN_3<br>or an other comparable commercial product	Merck	1.06549.0100

Working solutions / buffer

PBS/FCS-buffer	IsoFlow + 0,1% NaN_3 + 2 % FCS
1x Versa Lyse-Fix-solution	10 ml Versa-Lyse + 250 µl IOTest 3 Fixative solution (10x)

Antibodies and pipetting volumes

Antibody Panel	Company	Volume per test
BM01	Beckman Coulter	40 µl (20µl vial1 + 10µl vial2 + 10µl vial3)
BM02	Beckman Coulter	40 µl (20µl vial1 + 10µl vial2 + 10µl vial3)
BM03	Beckman Coulter	40 µl (20µl vial1 + 10µl vial2 + 10µl vial3)
BM04	Beckman Coulter	40 µl (20µl vial1 + 10µl vial2 + 10µl vial3)
BM06	Beckman Coulter	40 µl (20µl vial1 + 10µl vial2 + 10µl vial3)

Material for analysis

**Material**	EDTA whole blood
**Storing conditions**	transport at room temperature, storing in the laboratory at 4 °C
**Storage times**	max. 4h after blood collection, max 12h after end of staining

Sample preparation

01. EDTA-whole blood store within refrigerator, mix well prior to use
02. Label four 5ml flow cytometry tubes with „ONE 01 to ONE 04“ and patient ID
03. Label two 5ml flow cytometry tubes with “ONE 06” and patient ID
04. Add 100 µl EDTA-blood per tube

Surface staining

05. Add antibody mixes according to label on tube
06. Vortex for 10 s
07. Incubate for 15 min, RT (room temperature), in the dark
08. Vortex for 10 s
09. Add 1,5 ml 1x Versa-Lyse-Fix-solution to all tubes
10. Vortex briefly
11. Incubate for 15 min, RT (room temperature), in the dark
12. Vortex for 5 s
13. Add 2,5 ml cold PBS/FCS-buffer to all tubes
14. Centrifuge, 300 g, 5 min, 4 °C
15. Decant
16. Vortex for 5 s
17. Add cell suspension from one tube “ONE 06” to the second tube “ONE 06”
18. Add 3 ml PBS/FCS to all tubes
19. Centrifuge, 300 g, 5 min, 4 °C
20. Decant
21. Vortex for 5 s
22. Add 150 µl PBS/FCS-buffer (resulting in final volume about 250 µl)

Store at 4 °C in the dark until measurement

Setting information for NAVIOS cytometer

**Protocol:**	BM01	(for the stained blood sample tube BM01)
	BM02	(for the stained blood sample tube BM02)
	BM03	(for the stained blood sample tube BM03)
	BM04	(for the stained blood sample tube BM04)
	BM05	(for the stained blood sample tube BM05)

Cytosettings: ONE study Panel 01_2012 settings (for all tubes)

Sample designation:
ID1: Donor ID
ID2: Study
ID3: Panel
ID4: Analyst (three letter code: 1st letter “name” + 1st & 2nd letter “last name”)

Example: R5001V01_ONE-Study_BM01_MST

Supplemental Material

TDS VersaLyse Beckman Coulter
TDS IOTest3 Fixative Solution Beckman Coulter
TDS IsoFlow Beckman Coulter
