# Supplementary material for: Standardization of whole blood immune phenotype monitoring for clinical trials: panels and methods from the ONE study
Source: Transplant Res. 2013 Oct 25;2:17. doi: 10.1186/2047-1440-2-17 (PMC3827923; doi:10.1186/2047-1440-2-17)
Supplement: Additional file 3: Method S7 — Describing the standard operating procedure for staining of whole blood leukocytes with panel ONE 05. [file 2047-1440-2-17-S3.pdf]

Instruments

NAVIOS, Beckman Coulter
KALUZA analysis software, Beckman Coulter
Centrifuge
Vortexer
Test tube rotator
Refrigerator
Pipettes
Pipette boy

Material

Pipette tips (5 µl - 1000 µl) for low volume (µl) pipettes
Pipette tips for pipette boy
50 ml centrifuge tubes (e. g. Falcon™)
12x75mm-flow cytometry tubes
Rack for 12x75mm-tubes and 50 ml tubes

Name	Company	Order no.
IsoFlow™	Beckman Coulter	8546859
Red Blood Cell Lysis Solution (10X)<br>**or** Lysing Solution IOTest3 (10X)	Miltenyi<br>Beckman Coulter	130-094-183<br>A07799
IOTest 3 Fixative Solution (10X)	Beckman Coulter	A07800
Test Tube, 12x75mm Blue, 250 pieces	Beckman Coulter	2523749
FCS – head inactivated<br>or an other comparable commercial product	Biochrom	S0115
NaN_3<br>or an other comparable commercial product	Merck	1.06549.0100
Ampuwa<br>or an other comparable commercial product	Fresenius Kabi	

Working solutions / buffer

PBS/FCS-buffer	IsoFlow + 0,1% NaN_3 + 2 % FCS
1X Lysis-buffer	1,5 ml Red Blood Cell Lysing Solution (10x) + 13,5 ml Ampuwa<br>**or** 1,5 ml Lysing Solution IOTest3 (10x) + 13,5 ml Ampuwa
PBS-Fix-buffer	1ml IsoFlow + 25 µl IOTest 3 Fixative Solution (10x)

Antibodies and pipetting volumes

Antibody Panel	Company	Volume per test
BM05	Beckman Coulter	40 µl (20µl vial1 + 10µl vial2 + 10µl vial3)

Material for analysis

**Material**	EDTA whole blood
**Storing conditions**	Transport at room temperature, storing in the laboratory at 4 °C
**Storage times**	Max. 4h after blood collection, max 12h after end of staining

Please note: Problems with the lysis of the blood sample can follow an incomplete load of the EDTA blood collection tube with whole blood during the blood sampling procedure!!

Sample preparation

01. EDTA-whole blood store within refrigerator, mix well prior to use
02. Label two 5ml flow cytometry tubes with „ONE 05“ and patient ID
03. Label one 50 ml Falcon™ tube with patient ID
04. Add 15 ml 1X Lysis-buffer into the 50 ml Falcon™-tube
05. Inject 300 µl whole blood
06. Vortex
07. Incubate for 12 min, at RT (room temperature) on test tube rotator
08. Add 10 ml cold IsoFlow
09. Centrifuge, 300g, 10 min, 4 °C
10. Decant supernatant
11. Resuspend pellet
12. Add 300 µl cold IsoFlow
13. Transfer the suspension into one of the 5 ml-flow cytometry tubes
14. Add 4 ml cold IsoFlow
15. Centrifuge, 300g, 5 min, 4 °C
16. Decant supernatant
17. Resuspend pellets in rest volume
18. Transfer 100 µl cell suspension to the second labelled flow cytometry tube (If there is less volume then 100 µl volume adjust by adding IsoFlow buffer)

Please note: In case of an incomplete lysis of the whole blood sample (dark-red cell pellet) please repeat!! An incomplete lysed sample is not stainable!

Surface staining

19. Add antibody mixes according to label on the tube
20. Vortex for 10 s
21. Incubate 20 min, 4 °C, in the dark
22. Vortex for 10 s
23. Add 1 ml PBS-Fix buffer
24. Vortex briefly
25. Incubate 10 min, RT (room temperature), in the dark
26. Vortex for 5 s
27. Add 2 ml cold PBS/FCS-buffer
28. Centrifuge at 300 g, 5 min, 4 °C
29. Decant supernatant
30. Vortex for 5 s
31. Add 150 µl PBS/FCS-buffer (resulting in final volume 250 µl)

Store at 4 °C in the dark until measurement

Setting information for NAVIOS cytometer

Protocol: BM05 (for the stained blood sample tube BM05)
Cytosettings: ONE study Panel 05_2012 settings

Sample designation: ID1: Donor ID
ID2: Study
ID3: Panel
ID4: Analyst (three letter code: 1st letter "name" + 1st & 2nd letter "last name")

Example: R5001V01_ONE-Study_BM05_MST

Supplemental Material

TDS IOTest3 Fixative Solution Beckman Coulter
TDS Lysing Solution IOTest3 10X Beckman Coulter
TDS Red Blood Cell Lysis 10X Miltenyi
TDS IsoFlow Beckman Coulter
