# Supplementary figures and images for: Standardization of whole blood immune phenotype monitoring for clinical trials: panels and methods from the ONE study
Source: Transplant Res. 2013 Oct 25;2:17. doi: 10.1186/2047-1440-2-17 (PMC3827923; doi:10.1186/2047-1440-2-17)

Supplementary  
Figure 2

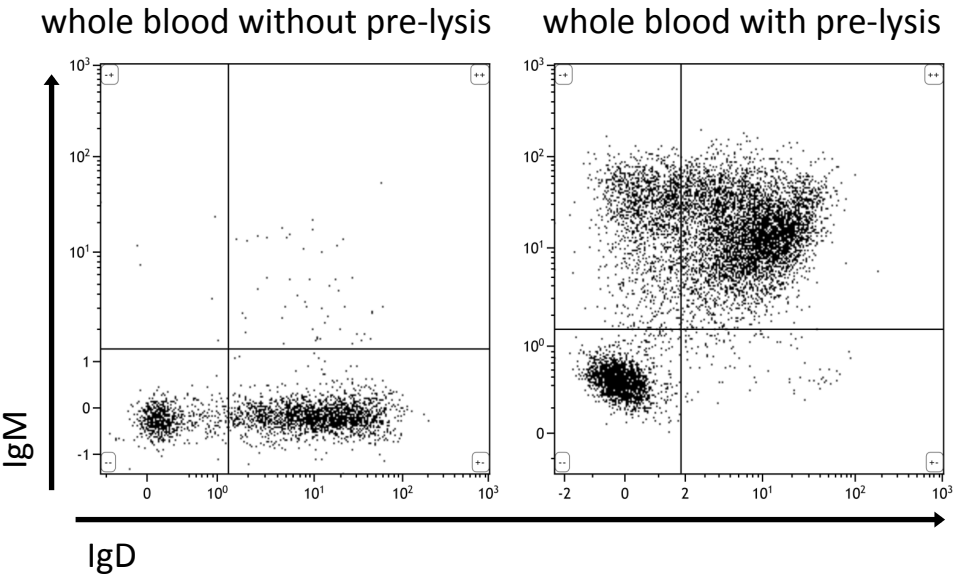

Supplement: Additional file 4: Figure S2 — Exemplary dot plots for surface IgM staining on CD19+ B cells comparing whole blood (WB) staining with and without prior removal of free plasma immunoglobulins by an ammonium chloride-based lyse/wash step. [file 2047-1440-2-17-S4.pdf]

Supplementary  
Figure 3

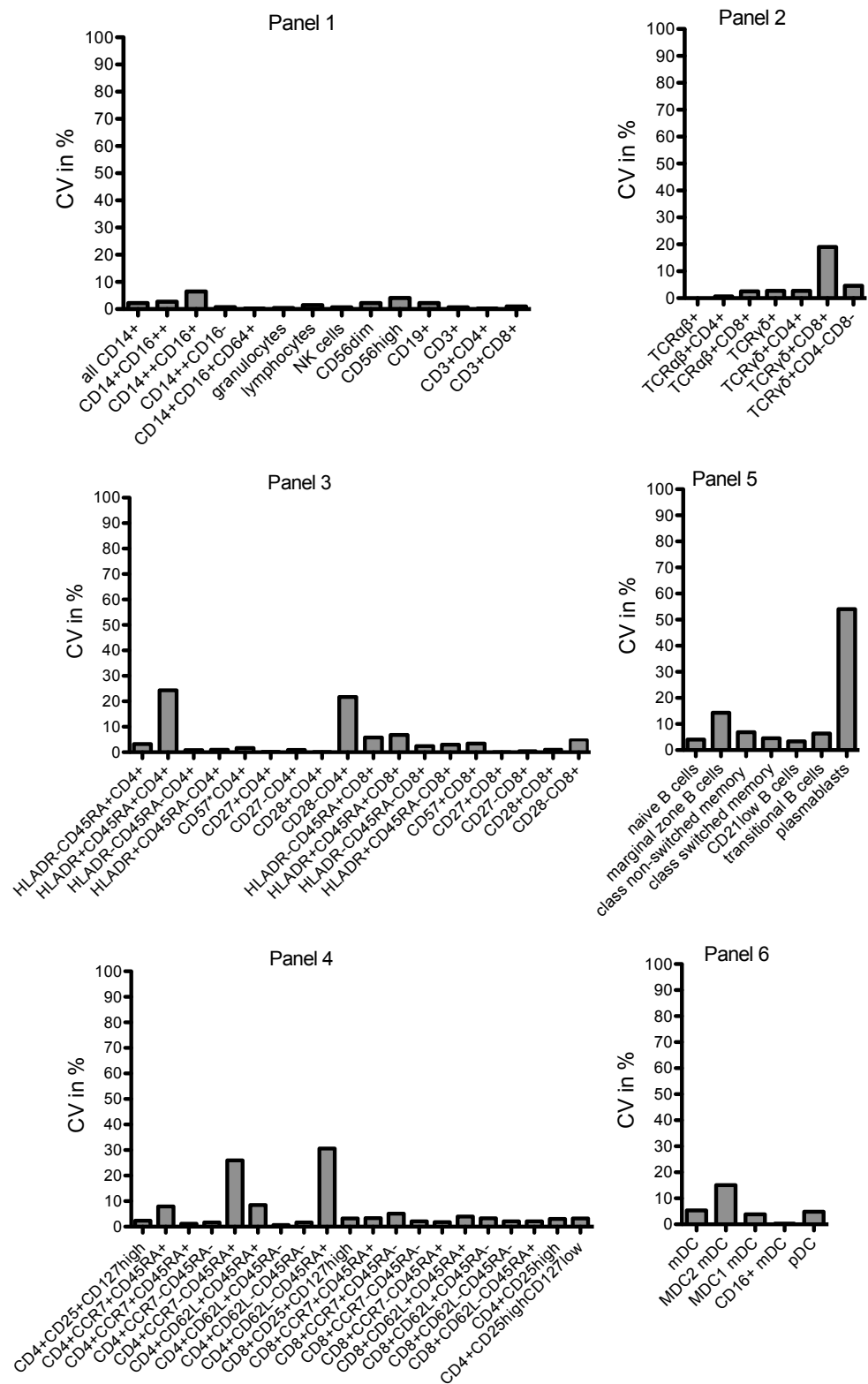

Supplement: Additional file 5: Figure S3 — Mean CVs of cell subsets were calculated in inter-operator test on samples collected from transplant patients 3 to 6 months after kidney transplantation for all six panels: panel ONE 01, general immune status; panel ONE 02, T cell subsets/αβ+ T cells and γδ+ T-cells; panel ONE 03, T cell activation; panel ONE 04, T cell memory and regulatory T cells; panel ONE 05, B cell subsets; and panel ONE 06, dendritic cell (DC) subsets. [file 2047-1440-2-17-S5.pdf]

Supplementary  
Figure 4

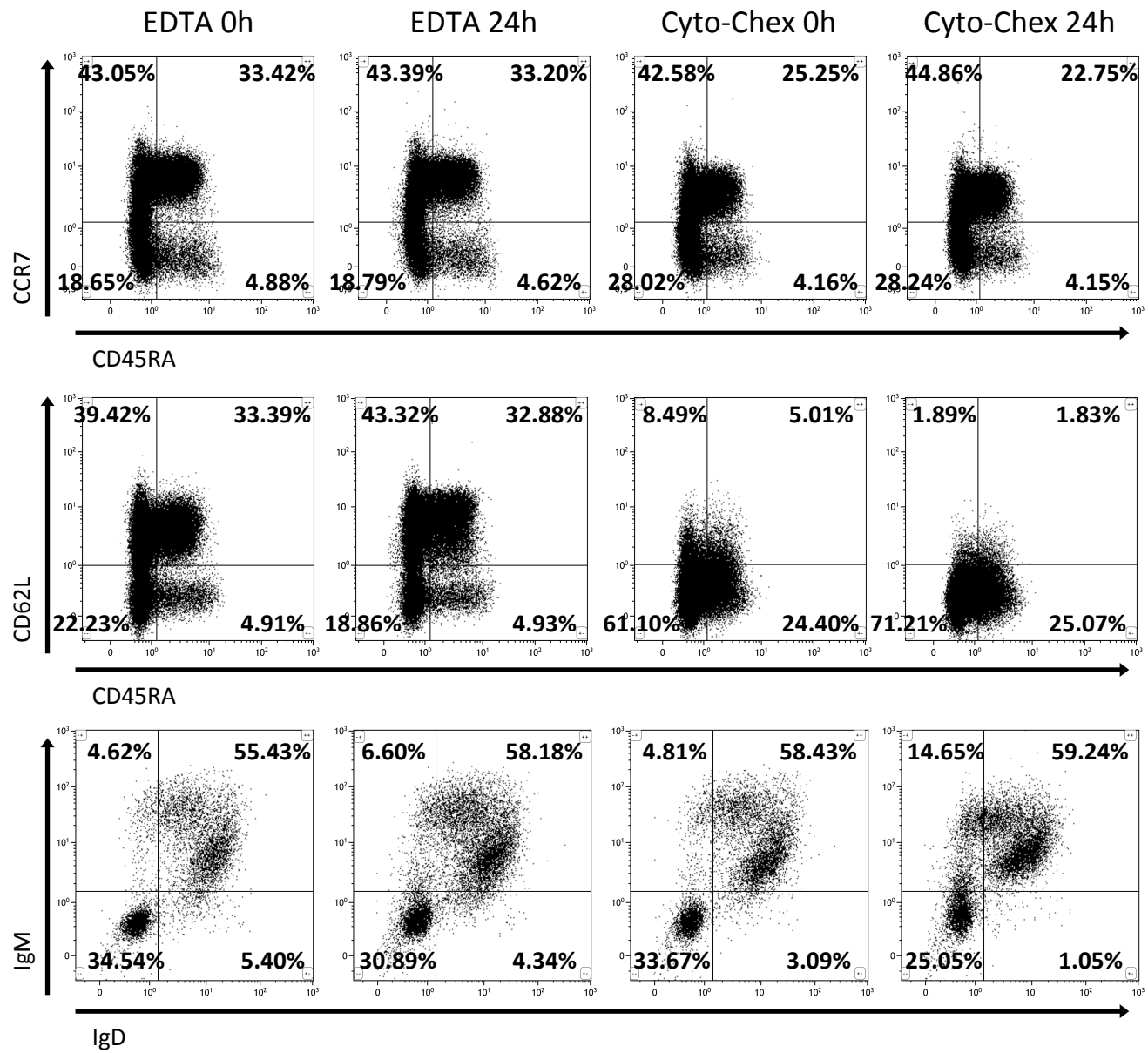

Supplement: Additional file 6: Figure S4 — Comparative analysis of leukocyte staining of whole blood (WB) samples collected into EDTA or Cyto-Chex tubes. Shown are dot plots of CCR7 versus CD45RA, and CD62L versus CD45RA staining for CD4+ T cells using the same gating strategy as described in Figure 5. Additionally IgM versus IgD staining of CD19+ B cells is displayed applying the same gating strategy as described in Figure 6. [file 2047-1440-2-17-S6.pdf]
