# Supplementary material for: Standardization of whole blood immune phenotype monitoring for clinical trials: panels and methods from the ONE study
Source: Transplant Res. 2013 Oct 25;2:17. doi: 10.1186/2047-1440-2-17 (PMC3827923; doi:10.1186/2047-1440-2-17)
Supplement: Additional file 7: Figure S5 — Shown are all results for the validation of the flow cytometry immune monitoring for the ONE Study, including all single CVs and mean CVs, respectively, and all changes from baseline and mean changes from baseline for all test assays. [file 2047-1440-2-17-S7.pdf]

Supplementary Figure 5

	intra-assay			inter-operator							inter-assay				
	sample Intr 01	sample Intr 02	mean	sample IO 01	sample IO 02	sample IO 03	sample IO 04	sample IO 05	sample IO 06	mean	sample inter 01	sample inter 02	sample inter 03	sample inter 04	mean
**subsets**	CV	CV	CV	CV	CV	CV	CV	CV	CV	CV	CV	CV	CV	CV	CV
Monocytes	0.06	0.09	7.25	0.26	0.10	0.11	0.14	0.19	0.10	14.97	0.26	0.15	0.19	0.11	17.98
CD14+ CD16high Monocytes	0.08	0.05	6.47	0.22	0.07	0.15	0.04	0.10	0.03	10.24	0.12	0.21	0.15	0.27	18.57
CD14high CD16+ Monocytes	0.18	0.09	13.45	0.51	0.29	0.46	0.56	0.10	0.26	36.11	0.65	0.60	0.17	0.07	37.33
CD14high CD16- Monocytes	0.09	0.10	9.19	0.28	0.01	0.38	0.15	0.11	0.15	18.11	0.15	0.36	0.13	0.03	16.77
CD14+ CD16+ CD64+ Monocytes	0.18	0.09	13.74	0.49	0.20	0.45	0.56	0.09	0.25	34.11	0.64	0.57	0.18	0.12	37.49
Granulocytes	0.06	0.06	5.64	0.08	0.00	0.01	0.00	0.10	0.03	3.71	0.22	0.16	0.13	0.15	16.43
Lymphocytes	0.04	0.05	4.13	0.02	0.01	0.01	0.00	0.14	0.02	3.56	0.17	0.20	0.13	0.11	15.40
NK cells	0.04	0.03	3.15	0.01	0.02	0.03	0.01	0.06	0.00	2.08	0.21	0.19	0.23	0.11	18.74
CD56dim NK cells	0.00	0.01	0.42	0.00	0.00	0.00	0.00	0.00	0.00	0.17	0.01	0.01	0.01	0.01	1.00
CD56high NK cells	0.05	0.04	4.08	0.04	0.01	0.02	0.05	0.05	0.18	5.78	0.33	0.32	0.31	0.16	28.10
B cells	0.02	0.03	2.58	0.03	0.03	0.02	0.03	0.05	0.03	3.38	0.11	0.09	0.09	0.13	10.34
T cells	0.00	0.00	0.39	0.00	0.00	0.00	0.00	0.01	0.00	0.43	0.02	0.03	0.02	0.02	2.36
CD4 T cells	0.01	0.01	0.90	0.00	0.00	0.01	0.00	0.01	0.01	0.73	0.03	0.02	0.01	0.06	3.18
CD8 T cells	0.02	0.02	1.76	0.00	0.00	0.03	0.00	0.00	0.01	0.84	0.06	0.01	0.01	0.04	3.04
TCRab T cells	0.00	0.00	0.13	0.00	0.00	0.00	0.00	0.00	0.00	0.07	0.00	0.00	0.00	0.00	0.27
TCRab CD4 T cells	0.00	0.01	0.49	0.01	0.00	0.00	0.00	0.01	0.01	0.52	0.03	0.01	0.01	0.03	2.26
TCRab CD8 T cells	0.01	0.02	1.44	0.01	0.01	0.01	0.00	0.01	0.01	0.80	0.07	0.02	0.01	0.02	3.05
TCRgd T cells	0.06	0.06	6.07	0.01	0.02	0.04	0.04	0.02	0.05	2.97	0.18	0.11	0.08	0.02	9.59
TCRgd CD4 T cells	0.26	0.37	31.55	0.38	0.01	0.48	0.24	0.24	0.26	26.79	0.63	0.54	0.26	0.18	40.10
TCRgd CD8 T cells	0.14	0.14	13.71	0.01	0.01	0.28	0.18	0.14	0.30	15.32	0.32	0.27	0.08	0.07	18.47
TCRgd CD4negCD8neg T cells	0.07	0.08	7.38	0.03	0.04	0.03	0.01	0.04	0.09	4.09	0.22	0.12	0.08	0.06	12.01
HLA-DRneg CD45RApos CD4 T cel	0.01	0.01	1.37	0.02	0.01	0.01	0.01	0.01	0.02	1.33	0.01	0.04	0.07	0.09	5.28
HLA-DRpos CD45RApos CD4 T cel	0.06	0.10	8.06	0.32	0.04	0.22	0.51	0.05	0.07	19.83	0.37	0.29	0.02	0.29	24.25
HLA-DRneg CD45RAneg CD4 T cel	0.01	0.01	1.02	0.01	0.00	0.01	0.02	0.01	0.00	0.89	0.01	0.01	0.06	0.01	1.99
HLA-DRpos CD45RAneg CD4 T cel	0.03	0.03	3.39	0.09	0.03	0.06	0.07	0.01	0.08	5.84	0.06	0.09	0.07	0.10	8.34
CD57pos CD4 T cells	0.07	0.07	7.09	0.08	0.01	0.04	0.08	0.00	0.21	7.06	0.06	0.23	0.13	0.16	14.69
CD27pos CD4 T cells	0.00	0.00	0.17	0.00	0.00	0.00	0.00	0.00	0.01	0.24	0.00	0.01	0.02	0.03	1.26
CD27neg CD4 T cells	0.06	0.06	5.99	0.02	0.01	0.03	0.01	0.01	0.06	2.25	0.06	0.05	0.15	0.10	9.19
CD28pos CD4 T cells	0.00	0.00	0.05	0.00	0.00	0.00	0.00	0.00	0.00	0.15	0.00	0.00	0.01	0.02	0.70
CD28neg CD4 T cells	0.12	0.20	15.54	0.08	0.01	0.08	0.38	0.04	0.23	13.72	0.42	0.29	0.18	0.18	26.95
HLA-DRneg CD45RApos CD8 Tcell	0.01	0.02	1.16	0.05	0.00	0.02	0.02	0.01	0.01	1.88	0.07	0.06	0.05	0.05	5.70
HLA-DRpos CD45RApos CD8 Tcell	0.03	0.02	2.49	0.13	0.02	0.00	0.07	0.05	0.02	4.75	0.09	0.15	0.04	0.05	8.27
HLA-DRneg CD45RAneg CD8 Tcell	0.02	0.01	1.42	0.02	0.01	0.02	0.01	0.07	0.02	2.48	0.14	0.09	0.05	0.06	8.34
HLA-DRpos CD45RAneg CD8 Tcell	0.07	0.02	4.36	0.11	0.02	0.02	0.07	0.02	0.05	4.88	0.07	0.06	0.06	0.03	5.30
CD57pos CD8 Tcells	0.04	0.04	4.32	0.04	0.04	0.02	0.08	0.02	0.04	4.08	0.15	0.11	0.08	0.03	9.27
CD27pos CD8 Tcells	0.00	0.00	0.28	0.01	0.01	0.00	0.00	0.03	0.01	0.99	0.01	0.03	0.04	0.06	3.38
CD27neg CD8 Tcells	0.06	0.02	3.92	0.03	0.03	0.01	0.06	0.08	0.04	4.09	0.15	0.09	0.17	0.04	11.10
CD28pos CD8 Tcells	0.00	0.01	0.42	0.01	0.01	0.00	0.01	0.01	0.02	1.03	0.02	0.05	0.05	0.08	4.84
CD28neg CD8 Tcells	0.03	0.02	2.36	0.03	0.04	0.01	0.08	0.02	0.04	3.51	0.17	0.11	0.08	0.04	9.66
CD25pos CD127high CD4 Tcells	0.01	0.03	2.07	0.09	0.10	0.05	0.03	0.05	0.03	5.57	0.03	0.07	0.09	0.02	5.23
CCR7pos CD45RApos CD4 Tcells	0.02	0.01	1.67	0.07	0.01	0.02	0.01	0.03	0.01	2.31	0.09	0.07	0.04	0.04	6.16
CCR7pos CD45RAneg CD4 Tcells	0.01	0.01	1.25	0.00	0.04	0.00	0.01	0.01	0.03	1.52	0.02	0.05	0.06	0.06	4.70
CCR7neg CD45RAneg CD4 Tcells	0.07	0.05	6.07	0.05	0.05	0.09	0.07	0.03	0.05	5.42	0.29	0.15	0.07	0.05	14.21
CCR7neg CD45RApos CD4 Tcells	0.10	0.08	9.34	0.10	0.33	0.09	0.05	0.04	0.06	11.08	0.64	0.52	0.17	0.13	36.37
CD62Lpos CD45RApos CD4 Tcells	0.02	0.02	1.70	0.06	0.01	0.02	0.01	0.03	0.00	2.11	0.05	0.05	0.05	0.03	4.55
CD62Lpos CD45RAneg CD4 Tcells	0.02	0.01	1.73	0.03	0.00	0.02	0.00	0.01	0.01	1.17	0.11	0.07	0.03	0.02	5.82
CD62Lneg CD45RAneg CD4 Tcells	0.03	0.03	3.09	0.00	0.01	0.04	0.06	0.05	0.00	2.81	0.08	0.05	0.10	0.02	6.12
CD62Lneg CD45RApos CD4 Tcells	0.10	0.21	15.36	0.08	0.00	0.09	0.05	0.11	0.02	5.75	0.59	0.15	0.16	0.19	27.19
CD25pos CD127high CD8 Tcells	0.09	0.06	7.58	0.19	0.02	0.10	0.14	0.07	0.03	9.14	0.25	0.07	0.31	0.04	16.81
CCR7pos CD45RApos CD8 Tcells	0.02	0.04	2.97	0.00	0.02	0.03	0.04	0.00	0.02	1.86	0.12	0.07	0.08	0.05	7.99
CCR7pos CD45RAneg CD8 Tcells	0.07	0.02	4.29	0.07	0.05	0.05	0.09	0.06	0.01	5.56	0.27	0.05	0.10	0.15	14.28
CCR7neg CD45RAneg CD8 Tcells	0.08	0.04	5.73	0.04	0.02	0.05	0.12	0.02	0.02	4.45	0.24	0.11	0.06	0.05	11.45
CCR7neg CD45RApos CD8 Tcells	0.08	0.03	5.81	0.07	0.10	0.06	0.04	0.01	0.02	5.05	0.21	0.09	0.11	0.01	10.74
CD62Lpos CD45RApos CD8 Tcells	0.02	0.05	3.19	0.02	0.04	0.03	0.03	0.00	0.01	2.05	0.09	0.03	0.10	0.07	7.26
CD62Lpos CD45RAneg CD8 Tcells	0.03	0.01	2.24	0.01	0.04	0.01	0.00	0.02	0.05	2.28	0.22	0.14	0.13	0.06	13.63
CD62Lneg CD45RAneg CD8 Tcells	0.02	0.01	1.91	0.01	0.03	0.02	0.05	0.00	0.00	2.02	0.10	0.05	0.06	0.02	5.50
CD62Lneg CD45RApos CD8 Tcells	0.07	0.03	5.14	0.01	0.01	0.07	0.02	0.01	0.01	1.99	0.18	0.17	0.15	0.08	14.69
CD25high CD4 Tcells	0.07	0.16	11.27	0.10	0.09	0.15	0.03	0.16	0.16	11.61	0.02	0.19	0.12	0.04	9.34
Treg CD4 Tcells	0.05	0.08	6.51	0.08	0.02	0.07	0.03	0.10	0.09	6.70	0.04	0.04	0.04	0.06	4.30
naive B cells	0.03	0.01	1.77	0.01	0.01	0.01	0.00	0.02	0.00	0.97	0.02	0.03	0.02	0.05	2.72
marginal zone B cells	0.04	0.12	8.08	0.05	0.03	0.07	0.01	0.10	0.04	4.94	0.11	0.12	0.16	0.08	11.79
class non switched B cells	0.05	0.05	5.12	0.04	0.06	0.03	0.01	0.04	0.01	3.24	0.18	0.05	0.12	0.06	10.16
class switched B cells	0.03	0.05	3.75	0.02	0.09	0.07	0.01	0.05	0.00	4.08	0.13	0.06	0.07	0.09	8.84
CD21low B cells	0.05	0.06	5.45	0.10	0.03	0.14	0.03	0.05	0.07	7.29	0.11	0.18	0.07	0.14	12.46
transitional B cells	0.18	0.09	13.56	0.02	0.03	0.32	0.01	0.11	0.12	10.26	0.19	0.15	0.25	0.15	18.57
Plasmablasts	0.31	0.23	27.26	0.29	0.04	0.22	0.15	0.09	0.20	16.57	0.33	0.17	0.32	0.31	28.19
mDC	0.06	0.03	4.42	0.06	0.03	0.01	0.10	0.07	0.02	4.64	0.25	0.24	0.13	0.05	16.57
MDC2 mDC	0.62	0.84	73.02	0.14	1.16	1.41	0.10	0.07	0.82	61.67	0.52	0.58	0.74	0.22	51.65
MDC1 mDC	0.12	0.17	14.54	0.23	0.19	1.41	0.33	0.16	0.22	42.24	0.28	0.38	0.10	0.08	20.82
CD16+ mDC	0.01	0.01	1.27	0.01	0.03	0.22	0.05	0.01	0.02	5.80	0.07	0.05	0.03	0.07	5.67
pDC	0.09	0.12	10.36	0.20	0.06	0.71	0.27	0.10	0.03	22.72	0.31	0.09	0.41	0.03	21.32

	Age-of-Blood 4 hours							Age-of-Blood 24 hours						
	sample AOB 01	sample AOB 02	sample AOB 03	sample AOB 04	sample AOB 05	sample AOB 06	mean	sample AOB 01	sample AOB 02	sample AOB 03	sample AOB 04	sample AOB 05	sample AOB 06	mean
**subsets**	Δ baseline	Δ baseline	Δ baseline	Δ baseline	Δ baseline	Δ baseline	Δ baseline	Δ baseline	Δ baseline	Δ baseline	Δ baseline	Δ baseline	Δ baseline	Δ baseline
Monocytes	0.18	42.41	11.59	25.54	23.98	13.70	19.57	33.05	99.85	19.58	63.52	43.11	64.30	53.90
CD14+ CD16high Monocytes	21.66	30.58	9.29	20.36	5.23	6.81	15.66	23.55	42.55	5.16	32.37	13.16	21.51	23.05
CD14high CD16+ Monocytes	67.39	10.02	17.20	32.97	14.16	5.72	24.58	10.27	221.28	64.48	0.09	0.36	3.87	50.06
CD14high CD16- Monocytes	15.08	12.22	5.23	34.85	13.12	7.32	14.64	2.37	11.91	8.14	7.00	3.04	8.61	6.85
CD14+ CD16+ CD64+ Monocytes	61.01	36.27	30.20	34.58	20.94	4.31	31.22	21.14	143.23	63.96	2.31	6.42	5.99	40.51
Granulocytes	5.00	28.91	9.62	0.63	1.61	21.77	11.26	4.26	22.57	0.34	8.56	6.63	10.30	8.78
Lymphocytes	5.65	14.36	5.14	0.73	2.17	10.45	6.42	23.84	25.20	18.25	25.92	10.96	20.72	20.82
NK cells	0.19	4.46	5.34	1.57	12.00	0.03	3.93	60.38	9.49	15.01	15.76	21.89	13.88	22.74
CD56dim NK cells	0.12	0.32	0.14	0.42	10.51	1.63	2.19	1.05	0.49	0.92	2.52	5.89	2.72	2.27
CD56high NK cells	3.16	10.45	3.69	3.13	29.43	15.52	10.90	28.69	15.90	24.87	18.85	16.50	25.96	21.80
B cells	5.96	5.03	4.60	8.57	3.90	0.08	4.69	26.67	7.19	18.83	36.00	10.08	20.02	19.80
T cells	1.12	0.65	0.21	0.99	1.40	0.10	0.75	13.07	4.14	4.73	8.96	0.29	4.64	5.97
CD4 T cells	0.13	3.42	0.29	2.06	0.46	0.80	1.19	18.82	8.10	1.20	7.89	3.30	0.95	6.71
CD8 T cells	1.11	8.00	0.75	6.48	0.01	2.72	3.18	24.36	17.07	0.82	20.17	5.18	2.78	11.73
TCRab T cells	0.50	0.06	0.23	1.24	0.30	0.11	0.41	1.05	0.50	0.46	3.61	1.22	0.56	1.23
TCRab CD4 T cells	0.37	0.30	1.61	2.21	0.38	0.34	0.87	18.56	5.74	3.11	5.92	2.63	2.48	6.41
TCRab CD8 T cells	0.71	0.48	2.53	3.68	0.04	1.95	1.57	23.78	9.54	2.04	6.22	1.99	7.89	8.58
TCRgd T cells	18.34	0.19	13.48	18.61	4.10	11.96	11.11	35.45	31.51	26.76	44.15	18.05	24.77	30.11
TCRgd CD4 T cells	59.51	36.24	8.88	27.31	50.40	68.71	41.84	100.00	100.00	11.60	0.21	35.13	35.63	47.09
TCRgd CD8 T cells	19.63	16.89	6.93	24.91	10.81	21.09	16.71	67.23	11.59	9.32	45.12	13.18	17.02	27.24
TCRgd CD4negCD8neg T cells	17.94	1.78	17.92	17.38	1.73	9.22	11.00	30.49	35.71	34.20	44.31	19.94	35.43	33.35
HLA-DRneg CD45RApos CD4 T cel	2.75	3.02	4.34	14.29	9.34	2.35	6.02	67.69	5.88	16.24	50.50	17.59	11.77	28.28
HLA-DRpos CD45RApos CD4 T cel	28.42	8.55	33.11	37.74	14.89	10.00	22.12	127.28	102.92	275.79	78.11	89.84	91.63	127.59
HLA-DRneg CD45RAneg CD4 T cel	0.10	4.58	1.44	1.30	0.49	4.25	2.03	35.15	6.97	8.14	5.70	4.92	17.40	13.05
HLA-DRpos CD45RAneg CD4 T cel	7.93	4.00	31.03	14.14	18.25	3.97	13.22	49.95	6.34	57.56	35.40	6.45	12.13	27.97
CD57pos CD4 T cells	24.75	20.52	10.33	8.57	9.11	12.75	14.34	26.09	24.34	34.34	65.89	5.27	62.58	36.42
CD27pos CD4 T cells	0.30	0.40	0.15	0.62	0.26	0.12	0.31	9.26	1.41	0.13	3.57	0.47	1.58	2.74
CD27neg CD4 T cells	2.35	14.22	3.51	7.02	4.11	2.32	5.59	72.80	50.34	3.09	40.20	7.35	30.02	33.97
CD28pos CD4 T cells	0.24	0.07	0.07	0.00	0.05	0.15	0.10	0.45	0.26	0.16	0.33	0.13	1.16	0.42
CD28neg CD4 T cells	28.50	103.12	11.98	2.65	17.59	8.70	28.75	52.82	392.38	29.11	217.67	49.37	66.66	134.67
HLA-DRneg CD45RApos CD8 Tcell	1.09	0.46	5.16	0.64	13.37	1.79	3.75	13.57	8.61	26.14	43.83	14.74	8.77	19.27
HLA-DRpos CD45RApos CD8 Tcell	5.28	6.01	15.09	25.50	8.57	2.38	10.47	25.03	3.24	56.51	18.47	1.25	42.35	24.47
HLA-DRneg CD45RAneg CD8 Tcell	0.12	8.57	1.41	1.88	3.03	11.17	4.36	54.58	15.99	18.92	21.24	17.77	32.37	26.81
HLA-DRpos CD45RAneg CD8 Tcell	4.93	12.88	26.60	11.20	23.39	0.11	13.19	20.01	8.53	79.10	40.21	9.56	16.89	29.05
CD57pos CD8 Tcells	3.21	5.10	0.33	0.65	5.15	16.41	5.14	29.25	6.55	29.13	34.18	7.14	24.72	21.83
CD27pos CD8 Tcells	1.34	0.38	0.46	2.71	2.64	0.32	1.31	21.76	1.56	1.82	9.09	3.31	0.97	6.42
CD27neg CD8 Tcells	4.48	10.19	6.99	32.88	30.13	7.03	15.28	72.45	41.35	27.89	110.35	37.79	21.08	51.82
CD28pos CD8 Tcells	1.45	1.18	0.41	0.03	1.61	0.68	0.89	16.38	1.99	2.23	5.90	2.32	1.17	5.00
CD28neg CD8 Tcells	4.14	9.68	3.01	0.14	5.89	13.32	6.03	46.83	16.28	16.31	30.03	8.50	22.87	23.47
CD25pos CD127high CD4 Tcells	4.16	6.96	4.93	7.99	9.08	25.76	9.81	11.90	27.89	8.93	25.29	8.32	4.89	14.54
CCR7pos CD45RApos CD4 Tcells	0.29	5.12	3.78	18.40	4.77	4.14	6.08	56.70	7.11	14.11	46.12	8.61	8.66	23.55
CCR7pos CD45RAneg CD4 Tcells	0.26	6.27	1.18	0.63	0.38	5.58	2.38	7.86	4.28	3.90	0.86	1.89	11.55	5.06
CCR7neg CD45RAneg CD4 Tcells	0.36	9.57	7.53	12.90	4.75	18.25	8.90	70.63	24.58	33.70	40.94	17.59	32.38	36.64
CCR7neg CD45RApos CD4 Tcells	3.16	26.07	28.49	23.05	14.87	2.10	16.29	96.69	55.97	34.51	2.26	4.02	33.94	37.90
CD62Lpos CD45RApos CD4 Tcells	0.54	4.52	3.33	18.07	5.23	4.43	6.02	60.46	5.96	14.04	46.13	10.12	10.70	24.57
CD62Lpos CD45RAneg CD4 Tcells	0.99	7.49	4.16	5.40	3.30	8.25	4.93	17.51	2.97	11.49	6.56	7.43	16.99	10.49
CD62Lneg CD45RAneg CD4 Tcells	2.51	5.37	1.76	2.74	4.42	7.38	4.03	65.95	31.83	9.71	17.05	12.63	10.38	24.59
CD62Lneg CD45RApos CD4 Tcells	1.28	73.91	18.70	53.57	38.60	14.05	33.35	15.29	200.77	203.60	136.27	202.39	119.01	146.22
CD25pos CD127high CD8 Tcells	2.40	22.16	16.81	25.31	7.49	15.07	14.87	33.44	46.34	25.29	39.10	36.95	23.44	34.09
CCR7pos CD45RApos CD8 Tcells	1.46	5.35	3.94	6.10	6.67	2.97	4.41	3.50	11.03	23.80	45.45	15.20	7.03	17.67
CCR7pos CD45RAneg CD8 Tcells	6.55	18.66	12.06	6.42	9.59	3.43	9.45	107.48	3.89	0.50	22.11	11.91	28.98	29.14
CCR7neg CD45RAneg CD8 Tcells	0.08	20.16	10.79	11.68	13.40	18.37	12.41	8.85	17.21	34.06	29.53	17.51	27.34	22.42
CCR7neg CD45RApos CD8 Tcells	0.67	4.75	6.72	15.82	2.25	5.79	6.00	45.73	1.34	31.95	12.08	9.03	7.63	17.96
CD62Lpos CD45RApos CD8 Tcells	4.16	3.68	2.78	5.49	4.63	2.80	3.92	12.09	10.64	20.68	43.76	12.31	8.84	18.05
CD62Lpos CD45RAneg CD8 Tcells	8.36	8.70	8.26	7.28	6.42	23.46	10.41	41.04	10.20	28.74	25.96	38.86	58.42	33.87
CD62Lneg CD45RAneg CD8 Tcells	5.44	3.96	2.29	2.63	4.20	4.68	3.87	12.16	18.82	22.05	27.46	0.84	7.84	14.86
CD62Lneg CD45RApos CD8 Tcells	10.85	14.92	1.29	24.07	3.95	12.14	11.20	30.85	2.71	26.24	15.93	18.27	53.86	24.64
CD25high CD4 Tcells	3.66	15.69	6.14	15.73	14.61	8.75	10.76	11.64	29.91	28.46	1.28	26.67	4.68	17.11
Treg CD4 Tcells	1.84	9.71	0.98	14.80	7.24	3.80	6.39	93.22	4.93	2.38	28.26	12.37	6.30	24.58
naive B cells	5.93	0.38	3.97	9.55	6.11	5.70	5.27	61.22	1.90	6.16	16.64	7.18	6.23	16.56
marginal zone B cells	3.58	5.36	13.19	13.21	7.09	13.15	9.26	60.47	1.74	14.03	22.37	1.03	18.61	19.71
class non switched B cells	5.88	29.03	0.69	11.48	17.20	3.67	11.32	26.01	33.48	11.38	6.30	10.19	11.61	16.49
class switched B cells	18.03	41.47	7.13	6.14	41.10	18.69	22.09	93.40	71.35	21.18	25.22	59.06	27.53	49.62
CD21low B cells	8.51	12.31	0.31	5.57	21.64	2.64	8.50	31.42	30.19	34.27	9.11	29.41	11.31	24.28
transitional B cells	83.83	18.62	7.20	7.55	36.60	47.70	33.58	416.93	30.03	37.50	33.37	26.54	86.76	105.19
Plasmablasts	39.38	60.29	31.70	40.71	45.83	62.47	46.73	82.69	88.59	29.37	14.47	45.07	116.24	62.74
mDC	23.14	10.63	1.94	4.77	15.88	2.04	9.73	26.00	13.00	10.08	5.80	22.31	64.69	23.65
MDC2 mDC	53.07	46.34	0.88	46.84	27.69	0.81	29.27	5.54	4.35	61.19	102.25	15.85	235.14	70.72
MDC1 mDC	13.81	30.43	19.95	24.64	8.72	25.60	20.53	65.30	11.71	33.05	20.96	0.81	51.09	30.49
CD16+ mDC	4.15	4.83	1.66	0.61	2.74	4.32	3.05	12.40	0.72	2.58	1.39	1.85	7.68	4.44
pDC	0.76	44.84	9.94	12.01	6.43	18.56	15.42	17.60	34.73	29.43	14.00	24.62	95.47	35.98

	AOS 4 hours					AOS 24 hours					inter-labortory-test				
	sample AOS 01	sample AOS 02	sample AOS 03	sample AOS 04	mean	sample AOS 01	sample AOS 02	sample AOS 03	sample AOS 04	mean	sample IL 01	sample IL 02	sample IL 05	sample IL 04	mean
**subsets**	Δ baseline	Δ baseline	Δ baseline	Δ baseline	Δ baseline	Δ baseline	Δ baseline	Δ baseline	Δ baseline	Δ baseline	CV	CV	CV	CV	CV
Monocytes	1.32	7.89	6.60	4.80	5.15	0.16	26.27	34.69	48.29	27.35	0.41	0.38	0.40	0.21	35.06
CD14+ CD16high Monocytes	22.68	8.61	14.75	2.23	12.07	15.79	32.92	9.07	0.71	14.62	0.24	0.13	0.25	0.56	29.46
CD14high CD16+ Monocytes	290.42	84.30	162.08	31.24	142.01	430.71	90.65	407.77	54.93	246.02	1.03	0.86	0.63	0.46	74.65
CD14high CD16- Monocytes	40.58	37.33	29.10	5.46	28.11	62.26	36.85	70.03	11.35	45.12	0.34	0.46	0.54	0.53	46.89
CD14+ CD16+ CD64+ Monocytes	275.70	77.68	151.23	23.53	132.04	420.22	79.34	378.28	48.63	231.62	1.05	0.89	0.68	0.48	77.63
Granulocytes	13.48	0.73	10.74	2.49	6.86	28.51	13.37	8.87	16.26	16.75	0.11	0.13	0.15	0.14	13.25
Lymphocytes	8.07	3.78	2.95	4.01	4.70	14.77	17.52	5.51	26.97	16.19	0.11	0.08	0.08	0.17	11.18
NK cells	3.61	0.72	2.80	2.52	2.41	1.27	8.30	15.27	6.35	7.80	0.16	0.02	0.05	0.09	8.00
CD56dim NK cells	0.22	0.01	1.96	0.76	0.74	0.43	0.54	1.04	1.49	0.87	0.01	0.01	0.00	0.01	0.70
CD56high NK cells	6.50	0.25	15.81	10.14	8.17	12.55	18.72	8.40	19.81	14.87	0.11	0.12	0.21	0.07	12.83
B cells	4.56	0.29	12.67	1.00	4.63	2.56	2.58	1.98	6.03	3.29	0.14	0.13	0.14	0.14	13.85
T cells	0.76	0.40	3.92	0.59	1.42	0.36	1.24	2.47	3.24	1.83	0.04	0.01	0.04	0.03	2.95
CD4 T cells	1.07	2.09	0.91	0.42	1.12	1.15	0.27	2.30	2.65	1.59	0.02	0.01	0.03	0.03	2.31
CD8 T cells	2.47	2.53	1.46	2.83	2.32	2.70	0.26	4.43	6.25	3.41	0.06	0.01	0.05	0.05	4.30
TCRab T cells	0.16	0.27	0.26	0.02	0.18	0.23	0.69	0.46	0.48	0.46	0.01	0.00	0.00	0.00	0.33
TCRab CD4 T cells	1.16	0.27	1.59	0.72	0.93	0.55	0.11	0.54	1.44	0.66	0.02	0.02	0.04	0.09	4.45
TCRab CD8 T cells	3.42	0.48	2.56	2.78	2.31	0.34	1.07	2.64	10.72	3.69	0.06	0.02	0.08	0.17	8.38
TCRgd T cells	1.51	3.10	7.58	0.87	3.26	13.35	21.26	13.55	20.90	17.26	0.15	0.13	0.08	0.11	11.53
TCRgd CD4 T cells	33.74	76.43	6.30	25.47	35.48	69.80	44.35	37.66	0.55	38.09	0.28	0.16	0.42	0.32	29.46
TCRgd CD8 T cells	4.33	28.80	27.72	21.51	20.59	40.55	63.94	17.20	2.88	31.14	0.23	0.16	0.13	0.10	15.21
TCRgd CD4negCD8neg T cells	3.34	1.56	5.54	11.57	5.50	23.08	17.23	11.14	30.13	20.40	0.10	0.14	0.06	0.12	10.77
HLA-DRneg CD45RApos CD4 T cel	0.65	2.59	1.11	4.96	2.33	3.41	1.09	15.76	17.82	9.52	0.15	0.05	0.02	0.08	7.49
HLA-DRpos CD45RApos CD4 T cel	56.79	4.95	14.51	9.83	21.52	424.18	80.36	124.38	229.03	214.49	0.25	0.48	0.50	0.54	44.49
HLA-DRneg CD45RAneg CD4 T cel	1.27	3.21	1.97	5.09	2.88	2.09	2.89	14.91	12.49	8.10	0.06	0.06	0.06	0.06	5.55
HLA-DRpos CD45RAneg CD4 T cel	4.73	6.83	12.30	7.44	7.82	1.08	16.37	2.43	8.53	7.10	0.20	0.12	0.15	0.08	13.75
CD57pos CD4 T cells	5.43	14.12	15.22	13.59	12.09	5.17	17.76	15.46	34.99	18.34	0.21	0.12	0.19	0.12	15.90
CD27pos CD4 T cells	0.32	0.43	0.01	0.52	0.32	0.52	0.65	1.47	3.32	1.49	0.02	0.01	0.00	0.01	0.94
CD27neg CD4 T cells	11.60	3.51	0.28	8.78	6.04	18.90	5.25	35.30	56.06	28.88	0.13	0.12	0.11	0.11	11.71
CD28pos CD4 T cells	0.00	0.12	0.01	0.03	0.04	0.03	0.38	0.02	0.12	0.14	0.01	0.00	0.00	0.00	0.25
CD28neg CD4 T cells	1.56	8.32	21.93	79.10	27.73	25.73	27.02	29.59	325.21	101.89	0.25	0.19	0.34	0.50	31.99
HLA-DRneg CD45RApos CD8 Tcell	1.20	2.79	0.29	4.39	2.17	5.25	5.86	7.21	4.28	5.65	0.15	0.09	0.12	0.19	13.65
HLA-DRpos CD45RApos CD8 Tcell	3.74	3.58	2.41	9.62	4.84	17.40	3.50	80.10	10.55	27.89	0.17	0.25	0.16	0.39	24.54
HLA-DRneg CD45RAneg CD8 Tcell	6.09	8.28	3.50	7.46	6.33	11.84	13.68	29.44	8.98	15.98	0.10	0.16	0.08	0.16	12.29
HLA-DRpos CD45RAneg CD8 Tcell	8.99	3.15	15.81	6.04	8.50	12.26	30.90	1.12	13.78	14.52	0.21	0.13	0.10	0.09	13.10
CD57pos CD8 Tcells	6.34	5.73	8.57	12.41	8.26	0.94	1.85	21.94	13.02	9.44	0.13	0.07	0.06	0.07	8.05
CD27pos CD8 Tcells	1.14	3.23	0.03	0.47	1.22	1.40	8.21	0.66	1.95	3.06	0.03	0.04	0.01	0.01	2.13
CD27neg CD8 Tcells	31.63	13.11	1.83	8.21	13.70	38.99	33.28	35.88	34.02	35.54	0.11	0.15	0.15	0.19	15.18
CD28pos CD8 Tcells	0.16	1.54	0.33	1.08	0.78	0.07	0.64	1.66	0.98	0.84	0.06	0.03	0.03	0.01	3.24
CD28neg CD8 Tcells	1.32	3.69	8.69	6.70	5.10	0.56	1.53	43.85	6.06	13.00	0.14	0.11	0.12	0.11	12.03
CD25pos CD127high CD4 Tcells	12.48	16.79	9.39	6.58	11.31	14.98	10.48	27.50	36.07	22.26	0.42	0.50	0.45	0.65	50.36
CCR7pos CD45RApos CD4 Tcells	1.17	4.68	1.30	3.16	2.58	2.33	6.18	9.89	10.36	7.19	0.17	0.05	0.03	0.10	8.82
CCR7pos CD45RAneg CD4 Tcells	0.30	9.09	1.96	6.36	4.43	4.01	2.55	0.90	3.48	2.74	0.09	0.07	0.08	0.13	9.33
CCR7neg CD45RAneg CD4 Tcells	14.01	5.61	0.70	3.97	6.07	27.74	7.53	26.02	37.03	24.58	0.33	0.23	0.51	0.65	43.16
CCR7neg CD45RApos CD4 Tcells	20.39	36.70	0.43	27.89	21.35	2.49	4.64	30.00	10.51	11.91	0.60	0.27	0.65	0.70	55.17
CD62Lpos CD45RApos CD4 Tcells	2.05	6.07	1.32	4.28	3.43	2.49	6.46	8.67	10.84	7.12	0.16	0.04	0.02	0.09	8.01
CD62Lpos CD45RAneg CD4 Tcells	3.41	6.59	1.11	1.87	3.24	1.09	11.59	3.51	0.59	4.19	0.06	0.04	0.08	0.05	5.78
CD62Lneg CD45RAneg CD4 Tcells	24.74	0.45	9.43	13.30	11.98	19.38	6.15	24.32	23.49	18.33	0.11	0.08	0.17	0.13	12.13
CD62Lneg CD45RApos CD4 Tcells	72.70	11.10	39.15	37.25	40.05	121.03	17.35	70.31	93.34	75.51	0.70	0.34	0.78	0.57	59.71
CD25pos CD127high CD8 Tcells	27.52	17.94	12.76	24.53	20.69	14.28	30.37	15.41	19.50	19.89	0.66	0.88	0.64	1.22	84.80
CCR7pos CD45RApos CD8 Tcells	2.86	2.71	0.01	2.24	1.95	0.70	1.08	4.09	21.47	6.83	0.18	0.06	0.16	0.14	13.29
CCR7pos CD45RAneg CD8 Tcells	10.46	12.13	3.86	17.72	11.04	9.14	16.91	18.53	20.45	16.26	0.16	0.16	0.28	0.55	28.83
CCR7neg CD45RAneg CD8 Tcells	15.07	1.00	2.54	4.05	5.67	6.36	4.11	7.36	15.26	8.27	0.29	0.12	0.37	0.47	31.22
CCR7neg CD45RApos CD8 Tcells	5.77	2.59	8.20	5.65	5.55	3.29	8.05	2.21	24.39	9.49	0.24	0.08	0.35	0.30	24.38
CD62Lpos CD45RApos CD8 Tcells	2.49	2.50	0.23	3.52	2.18	1.50	1.14	4.05	19.94	6.66	0.18	0.05	0.12	0.14	12.48
CD62Lpos CD45RAneg CD8 Tcells	22.26	20.49	0.65	5.23	12.16	45.76	29.99	18.35	40.93	33.76	0.06	0.04	0.08	0.17	8.52
CD62Lneg CD45RAneg CD8 Tcells	1.10	0.84	1.01	6.88	2.46	13.99	6.72	8.77	4.45	8.48	0.11	0.08	0.12	0.13	10.96
CD62Lneg CD45RApos CD8 Tcells	8.79	3.24	2.63	7.66	5.58	9.51	2.18	2.55	28.18	10.60	0.15	0.09	0.16	0.29	17.13
CD25high CD4 Tcells	32.21	28.16	20.88	17.73	24.74	30.09	32.18	42.90	37.42	35.65	0.32	0.56	0.41	0.80	52.31
Treg CD4 Tcells	15.78	2.83	4.45	5.01	7.02	12.46	3.07	11.92	5.45	8.22	0.07	0.13	0.15	0.48	20.60
naive B cells	0.79	4.22	0.61	3.44	2.26	2.30	4.34	7.68	1.68	4.00	0.08	0.07	0.03	0.08	6.46
marginal zone B cells	10.96	13.03	3.12	8.56	8.92	0.92	15.67	24.06	15.80	14.11	0.21	0.15	0.13	0.18	16.76
class non switched B cells	5.88	10.39	2.14	6.80	6.30	5.65	8.70	8.40	4.75	6.88	0.17	0.09	0.11	0.13	12.50
class switched B cells	0.89	0.65	9.25	11.58	5.59	6.43	7.60	25.63	9.38	12.26	0.27	0.12	0.81	0.17	34.30
CD21low B cells	1.90	24.83	3.40	0.57	7.67	12.62	18.60	30.01	3.23	16.12	0.33	0.18	0.21	0.31	25.45
transitional B cells	18.39	29.39	25.63	6.45	19.97	46.44	5.62	43.29	25.80	30.29	0.77	0.38	0.27	0.24	41.78
Plasmablasts	19.02	12.87	32.67	14.41	19.74	3.19	7.30	0.94	15.75	6.79	0.52	0.13	0.96	0.26	46.50
mDC	0.17	10.36	2.31	52.12	16.24	2.38	12.87	8.52	60.13	20.98	0.20	0.16	0.23	0.18	19.26
MDC2 mDC	100.00	240.94	41.31	5.47	96.93	100.00	100.00	16.72	61.90	69.65	1.39	1.03	0.87	0.94	105.51
MDC1 mDC	46.22	20.58	1.00	2.39	17.55	44.78	28.35	20.73	2.35	24.05	0.50	0.73	0.43	0.51	54.30
CD16+ mDC	9.45	2.33	4.56	0.84	4.30	9.79	4.07	13.44	5.25	8.14	0.03	0.11	0.04	0.03	5.31
pDC	67.13	15.53	18.38	16.29	29.33	80.07	11.06	26.15	43.05	40.08	0.28	0.42	0.43	0.12	31.06
